# Genetic structure and *Rickettsia* infection rates in *Ixodes ovatus* and *Haemaphysalis flava* ticks across different altitudes

**DOI:** 10.1371/journal.pone.0298656

**Published:** 2024-03-13

**Authors:** Maria Angenica F. Regilme, Megumi Sato, Tsutomu Tamura, Reiko Arai, Marcello Otake Sato, Sumire Ikeda, Kozo Watanabe

**Affiliations:** 1 Center for Marine Environmental Studies (CMES), Ehime University, Matsuyama, Ehime, Japan; 2 Graduate School of Science and Engineering, Ehime University, Matsuyama, Ehime, Japan; 3 Graduate School of Health Sciences, Niigata University, Niigata, Japan; 4 Niigata Prefectural Institute of Public Health and Environmental Sciences, Niigata, Japan; 5 Faculty of Medical Technology, Division of Global Environment Parasitology, Niigata University of Pharmacy and Medical and Life Sciences, Niigata, Japan; 6 Research Laboratories, Research and Development Headquarters, Earth Corporation, Hyogo, Japan; University of Bari, ITALY

## Abstract

Ixodid ticks, such as *Ixodes ovatus* and *Haemaphysalis flava*, are important vectors of tick-borne diseases in Japan, such as Japanese spotted fever caused by *Rickettsia japonica*. This study describes the *Rickettsia* infection rates influenced by the population genetic structure of *I*.*ovatus* and *H*. *flava* along an altitudinal gradient. A total of 346 adult *I*. *ovatus* and 243 *H*. *flava* were analyzed for the presence of *Rickettsia* by nested PCR targeting the 17kDA, *gltA*, *rOmpA*, and *rOmpB* genes. The population genetic structure was analyzed utilizing the mitochondrial cytochrome oxidase 1 (*cox1*) marker. The *Rickettsia* infection rates were 13.26% in *I*. *ovatus* and 6.17% in *H*. *flava*. For *I*. *ovatus*, the global *F*_ST_ value revealed significant genetic differentiation among the different populations, whereas *H*. *flava* showed non-significant genetic differentiation. The *cox1 I*. *ovatus* cluster dendrogram showed two cluster groups, while the haplotype network and phylogenetic tree showed three genetic groups. A significant difference was observed in *Rickettsia* infection rates and mean altitude per group between the two cluster groups and the three genetic groups identified within *I*. *ovatus*. No significant differences were found in the mean altitude or *Rickettsia* infection rates of *H*. *flava*. Our results suggest a potential correlation between the low gene flow in *I*. *ovatus* populations and the spatially heterogeneous *Rickettsia* infection rates observed along the altitudinal gradient. This information can be used in understanding the relationship between the tick vector, its pathogen, and environmental factors, such as altitude, and for the control of tick-borne diseases in Japan.

## Introduction

Tick-borne diseases are a significant public health concern in Japan and are transmitted by a diverse range of tick species, such as *Ixodes ovatus* [1] that potentially transmit *Borrelia sp*. causing *Lyme* disease [2] and *Haemaphysalis flava* [3], which transmits *Rickettsia japonica* and is a suspected vector of severe fever with thrombocytopenia syndrome virus [4–6]. Their dispersal is linked to the mobility of their hosts, relying on them to disperse into new landscapes and potentially introduce pathogens [7, 8]. The dynamics of tick-borne pathogens are influenced by the habitat distribution and dispersal behaviors of vectors and hosts along environmental gradients [9]. Therefore, understanding the complex interaction between these factors is important in understanding the spread of tick-borne diseases in Japan.

Tick population genetic analysis provides data that help identify the dispersal pattern of ticks based on gene flow between local populations [10]. The potential of spreading pathogens might be influenced by ticks’ dispersal, which is related to the movements of their vertebrate hosts, especially in three-host Ixodidae species [7, 8]. For example, contrasting patterns in the population genetic structures of *I*. *ovatus* and *H*. *flava* in the Niigata Prefecture of Japan suggest that host mobility during the immature stages of tick development may influence the genetic structure of adult ticks by affecting survivability into their adult stages [11, 12]. *Ixodes ovatus* populations had greater genetic divergence possibly due to the limited dispersal of their small mammalian hosts during the immature development stage; *H*. *flava* populations showed a more homogenized structure possibly due to the larger mobility of their large mammalian hosts and avian-mediated dispersal [11]. Other studies have also revealed low gene flow in ticks with low-mobility hosts (e.g., small mammals) and higher gene flow in ticks with highly mobile hosts (e.g., large mammals and birds) [10, 13–16].

The spatial distribution and movement of the vector (i.e., ticks) may determine the spatial distribution of the pathogen’s (i.e., *Rickettsia*) infection rate [17]. Previous studies have shown that the pathogen infection rate can be influenced by many factors, such as the vector’s genetic diversity, gene flow, and spatial structure [5, 6, 11, 18–25]. For example, previous studies have shown that strong gene flow between local vector populations tends to reduce the spatial heterogeneity of pathogen infection rates between populations [26, 27]. Thus, the spatial distribution and movement of the vector may affect the spatial distribution of the pathogen. To our knowledge, no previous studies have examined the relationship between the spatial heterogeneity of *Rickettsia* infection rates and population genetic structure in ticks.

Environmental factors may relate to the population genetic structure of ticks [28, 29], with limited gene flow increasing genetic variation between populations along an altitudinal gradient, as reported in several studies on other species [30–32]. In the study by [11], no significant influence of environmental factors, including altitude, was observed in the genetic structures of *I*. *ovatus* and *H*. *flava* based on the mantel test, but the study did not use any other robust analytical methods to thoroughly examine the influence of altitude on tick genetic structure. In another study, major spotted fever group *Rickettsia* (SFGR) prevalence was analyzed in a total of 3,336 immature and adult ticks across the Niigata Prefecture, Japan in the following tick species: *Dermacentor taiwanensis*, *H*. *flava*, *Haemaphysalis hystricis*, *Haemaphysalis longicornis*, *Haemaphysalis megaspinosa*, *Ixodes columnae*, *Ixodes monospinosus*, *Ixodes nipponensis*, *Ixodes ovatus*, and *Ixodes persulcatus* [6]. Three SFGR species namely *Rickettsia asiatica*, *R*. *helvetica* and *R*. *monacensis* were detected in *H*. *flava*, *Haemaphysalis longicornis*, *Ixodes monospinus*, *Ixodes nipponensis*, and *Ixodes ovatus*, no spatial distribution of *Rickettsia* infection rates was found among the local populations. To our knowledge, no previous studies have considered the influence of environmental factors on the spatial distribution of Spotted fever group *Rickettsia* infection rates along an altitudinal gradient in local Ixodid tick populations such as *Ixodes ovatus* and *Haemaphysalis flava* as influenced by the tick population’s genetic structure.

In this study, we elucidate the relationship between *Rickettsia* infection rates as influenced by population genetic structure along an altitudinal gradient to improve public health understanding of the distribution of ticks and tick-borne diseases. Based on the isolation by environment (IBE) theory, genetic differentiation increases with environmental variation, regardless of geographic distance [33–35]. Thus based on the results of [11], we hypothesized that in *I*. *ovatus* with a strong population genetic structure, we expect to see a heterogenous *Rickettsia* infection rate along an altitudinal gradient. In contrast to the homogenous genetic structure of *H*. *flava* wherein we expect to observe a homogenous *Rickettsia* infection rate.

## Materials and method

### Published data of [6, 11]

In this study, we used *cox1* sequence data from [11] for *I*. *ovatus* (n = 307) and *H*. *flava* (n = 220) ticks collected from April 2016 to November 2017 from 30 sites across the Niigata Prefecture, Japan. Sequences used for analysis are available in the GenBank database under the accession numbers MW063669-MW064124 and MW065821—MW066347. *Rickettsia* infection rate data were obtained from [6] from *I*. *ovatus* (n = 29) and *H*. *flava* (n = 2), from 38 sites across Niigata Prefecture. The 38 sites surveyed in the previous study by [6] include the 30 sites that were used in this present study (S1 Table). Please refer to [6, 11] for more information about the study sites, collection, sampling identification, DNA extraction, PCR amplification, and sequencing methods used in each respective study.

To strengthen our analysis, we also added new *cox1* sequences and *Rickettsia*-infected/uninfected ticks from *I*. *ovatus* (n = 39) and *H*. *flava* (n = 23) individuals collected from April to October 2018, a total of (n = 62) sampled at 30 sites across the Niigata Prefecture, including two sites not previously sampled by [11]. The sequences are available in the GenBank database under the accession numbers OR975837 to OR975875 and OR975876 to OR975898. At these sites, ticks were collected 2–14 times from six core sites among the 30 sites, while ticks were collected once at the remaining sites. The altitude at each site ranged from 8 to 1402 meters above sea level (m.a.s.l.), with a mean altitude of 348 m.a.s.l.

### Unpublished data from the 2018 collection

Ticks collected were stored at 4°C in microcentrifuge tubes with 70% ethanol. Each collected tick was morphologically identified using a stereo microscope following the identification keys of [36]. Genomic DNA was extracted from individual ticks using Isogenome DNA extraction kits (Nippon Gene Co. Ltd. Tokyo, Japan) following the manufacturer’s recommended protocol.

In this study, we combined previously published data from [6] with our newly collected data to calculate the *Rickettsia* infection rate, which is the percentage of *Rickettsia*-infected ticks from each obtained population. We analyzed the obtained tick DNA for spotted fever group *Rickettsia* (SFGR) detection and host identification and amplified the mitochondrial gene *cox1* for population genetic analysis. We performed nested PCR targeting the following genes for the detection of *Rickettsia* sp.: 17-kDA antigen gene (17-kDA); citrate synthase gene (gltA); spotted fever group (SFG)-specific outer membrane protein A gene (rOmpA); and outer membrane protein B gene (rOmpB) as described and analyzed in [6, 37–41] (S2 Table). Briefly, we first amplified the 17-kDa protein. If the results were positive, then PCR was performed to target gltA. Samples that were positive with both 17-kDA and gltA were regarded as positive for SFGR and a nested PCR was performed to target the rOmpA and rOmpB gene, samples that are positive for 17-Kda, gltA, rOmpA, and rOmpB genes were sequenced to identify the *Rickettsia* species. The amplified PCR products were purified using AMPure XP (Beckman Coulter Co., Japan) and sequenced using the Big Dye Terminator Cycle Sequence Kit (Thermo Fisher Scientific).

The *cox1* mitochondrial gene was amplified by PCR for *cox1* (658 base pairs) using the primer pairs LCO-1490 (5′-GGTCAACAAATCATAAAGATATTGG-3’) and HCO1–2198 (5′–AAACTTCAGGGTGACCAAAAAATCA-3) for phylogenetic analysis and tick species identification [42]. The PCR amplification profile included an initial denaturation of 94°C for 2 min, followed by denaturation at 94°C for 30 s, then annealing at 38°C for 30 s, followed by an extension of 72°C for 1 min for 30 cycles, and a final extension of 72°C for 10 min. The obtained PCR products were purified using the QIAquick 96 PCR Purification Kit (Qiagen, Germany) following the manufacturer’s instructions and were sequenced by Eurofin Genomics, Inc. (Tokyo, Japan).

Each forward and reverse read was assembled using CodonCode Aligner version 1.2.4 software (https://www.codoncode.com/aligner/). Low-quality bases were removed in the aligned sequences, and no ambiguous bases were detected. We used the MAFFT alignment online program (https://mafft.cbrc.jp/alignment/server/) to perform multiple alignments using the default settings. The sequences were checked for similarities with the deposited reference sequences from GenBank for sequence quality and tick species confirmation using BLAST (https://blast.ncbi.nlm.nih.gov/Blast.cgi). The protein-coding genes were translated to amino acids to confirm the absence of stop codons and the final aligned sequences were checked in Mesquite version 3.5 [43].

### Population genetic analysis

Multiple sites that are within 80 kilometers were combined for population genetic analysis if less than eight individuals were obtained per site, which resulted in 8 populations labeled A to H (S1 Table). Three sites were excluded from the population genetic analysis because of the limited number of obtained individuals (<8) and the lack of a nearby site within 80 kilometers to combine into a single population.

The final *cox1* sequences of the tick species: *I*. *ovatus* and *H*. *flava* were individually analyzed using DNASp version 6.12.03 to determine the haplotype diversity per species [44]. The level of genetic divergence between each population was quantified per species using global *F*_ST_. Significance was tested using Arlequin software version 3.5.2.2 [45] with 9999 permutations.

The genetic relationship between the *I*. *ovatus* populations was visualized using the unweighted pair group with the arithmetic mean (UPGMA) cluster method using the APE package [46] for the RStudio software (R Development Core Team, 2016). A cluster dendrogram was created using pairwise *F*_ST_ values genetic distance matrix from GenAlEx.

### Haplotype network and phylogenetic analyses

We constructed a haplotype network analysis using PopART program version 1.7 (http://popart.otago.ac.nz/index.shtml) on *cox1 I*. *ovatus* and *H*. *flava* sequences to assess haplotype relationships and the distribution of *Rickettsia* infected infected ticks using the median-joining network algorithm [47]. Briefly, we constructed a Bayesian phylogenetic tree of *cox1* haplotypes for *I*. *ovatus* and *H*. *flava*, respectively, using Markov chain Monte Carlo (MCMC) approach implemented in the BEAST version 1.10.14 [48]. We used the Hasegawa-Kishino-Yano substitution model with estimated base frequencies. We employed a strict clock model and used the coalescent prior as the tree prior. A maximum clade credibility tree was acquired using TreeAnnotator version 1.10.14 using trees from BEAUti version1.10.14 with 90% of the trees as the burn-in. We viewed the constructed maximum clade credibility tree using FigTree version 1.4.4.

### Statistical analysis

To determine whether there was a significant difference in the *Rickettsia* infection rate between haplotype groups for *I*. *ovatus* and *H*. *flava*, we performed a z-score test at p < 0.05. The z-score test was chosen because of the large sample size and because the population variance was known. To determine whether there were differences in the mean altitude between the haplotype groups, we used the Welch t-test at p < 0.05. Welch t-test was used when the means of the two populations were normally distributed and had equal variances.

## Results

The total number of positive (pos) and negative (neg) ticks for *Rickettsia* infections from the ticks collected in 2018 were: *I*. *ovatus* (neg = 22, pos = 17) and *H*. *flava* (neg = 10, pos = 13). In this study, the total number of samples from the previously published data [6, 11] and the unpublished data from 2018 were: *I*. *ovatus* (n = 346) and *H*. *flava* (n = 243). The number of adult ticks whose *cox1* was successfully sequenced per species were: *I*. *ovatus* (346) and *H*. *flava* (243) (Table 1).

**Table 1:** Summary of the haplotype and *Rickettsia* infection rates among the 7 ixodid tick species obtained in the Niigata Prefecture, Japan.

Tick species **	ns	n	nh	r	Global F_ST_
1. *Ixodes ovatus*	30	346	59	46 (13.26%)	0.4154*
2. *Haemaphysalis flava*	18	243	66	15 (6.17%)	0.3597
**Total**		589		61	

Abbreviations: ns no. of sampling sites; n sample size; nh no of haplotypes; r *Rickettsia* infection rate per species

*p < 0.05 **tick species identification is based on molecular identification using the *cox1* marker and BLAST results

We detected SFGR in 78 (12.44%) out of 627 ixodid ticks, with the highest detected in *I*. *ovatus* (46/346; 13.29%) and in *H*. *flava* (15/243; 6.17%) as summarized in Table 1. Out of the 46 *Rickettsia-*infected *I*. *ovatus* ticks, 25 displayed a 100% identity match with *Rickettsia asiatica* in the 17kDA, *gltA*, and *rOmpB* markers [6], while an additional 19 adult *I*. *ovatus* from the 2018 collection were also positive with *R*. *asiatica*. Two haplotypes were found in the rOmpB and 17kDA markers, respectively. One haplotype was found in only one individual (17369). Two out of the 15 *Rickettsia*-infected *H*. *flava* ticks were found to have the same haplotypes in the 17Kda, gltA,and rOmpA markers, and were identified as *Rickettsia sp*. (LC461063). The remaining 13 *Rickettsia*-infected *H*. *flava* ticks were identified as *Rickettsia sp*.

Based on the population genetic analysis of the *cox1* sequences, we found a significant global *F*_ST_ of 0.4154 at *p* < 0.05 for *I*. *ovatus* (Table 1). In contrast, no significant global *F*_ST_ values were observed in the *H*. *flava* (Table 1). There were 59 and 66 *cox1* haplotypes found among the 346 *I*. *ovatus* and 243 *H*. *flava* individuals, respectively.

**Fig 1:**
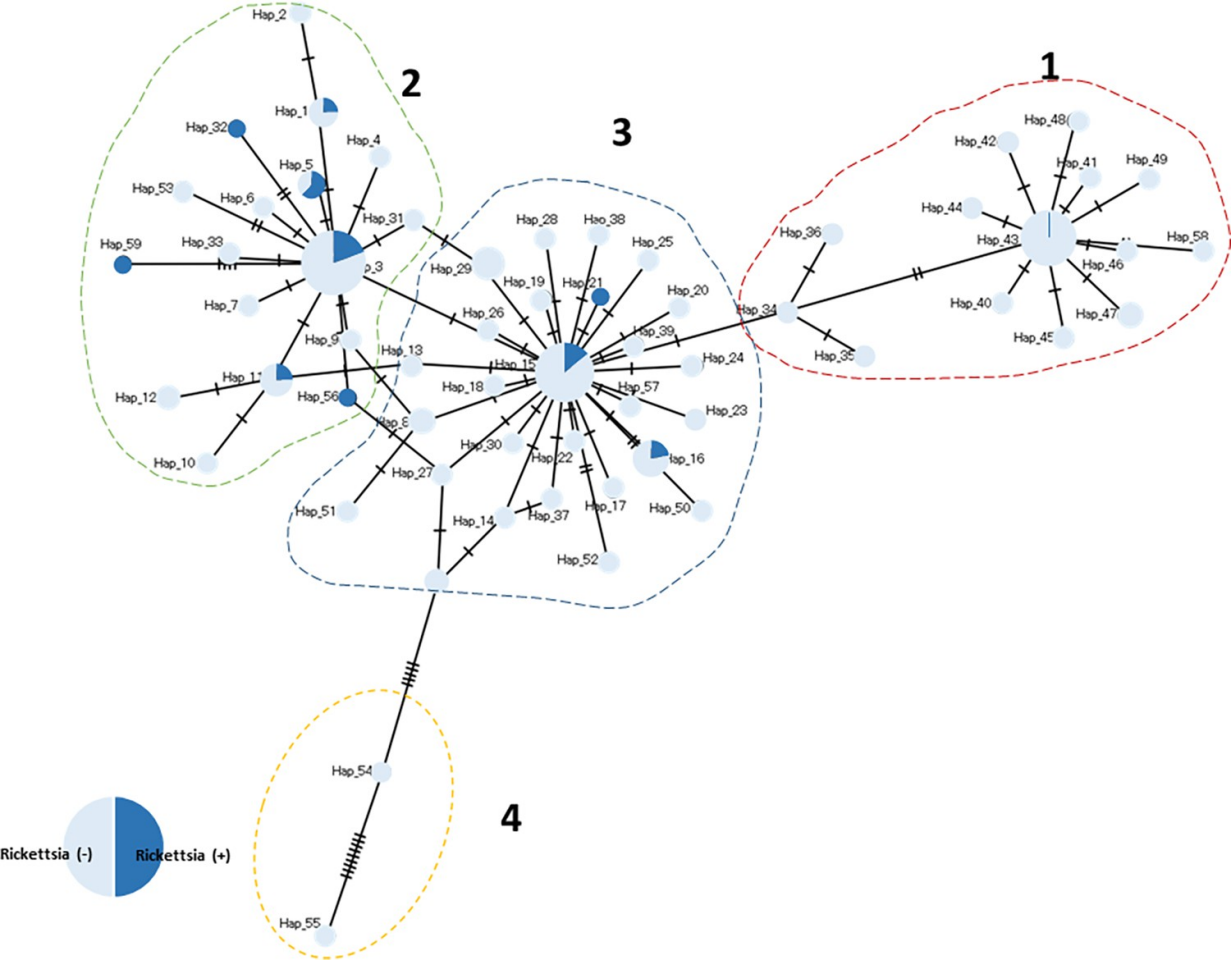
Median-joining network of the 59 *cox1* haplotype sequences of *Rickettsia* positive and negative *I*. *ovatus*. Haplotype groups are indicated by numbers (1 to 4).

**Fig 2:**
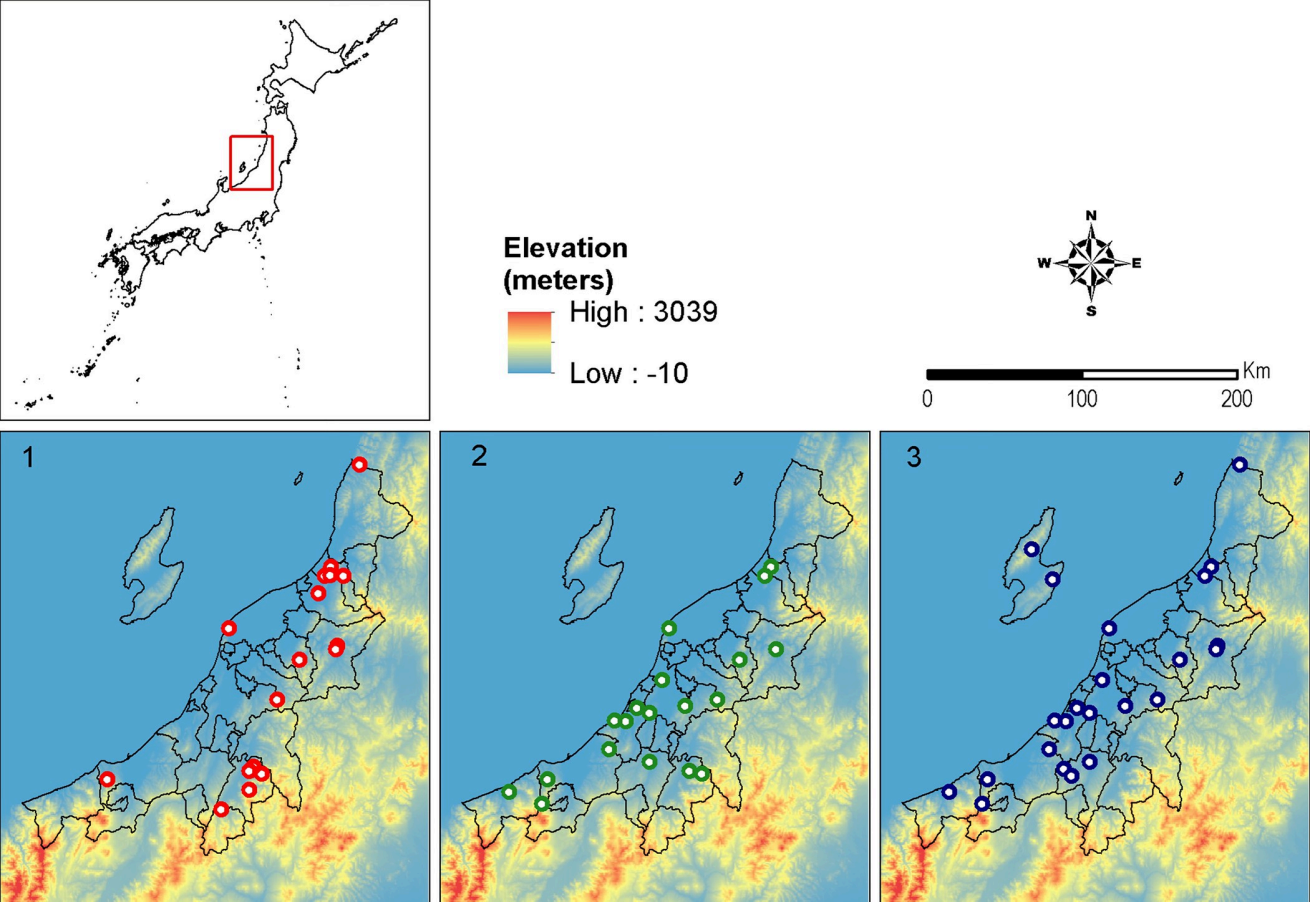
The influence of altitude on the habitat distribution of *I*. *ovatus*. The points indicate the sampling sites of the collected individuals per *I*. *ovatus* haplotype group (n = 3) in the haplotype network (Fig 1). The map shows the elevation level across the sampling area, with Niigata Prefecture Japan depicted as a color gradation. Group 4, not shown in this figure, with haplotypes 54 and 55 was found on Sado island, which is encircled in black in box 3.

**Fig 3:**
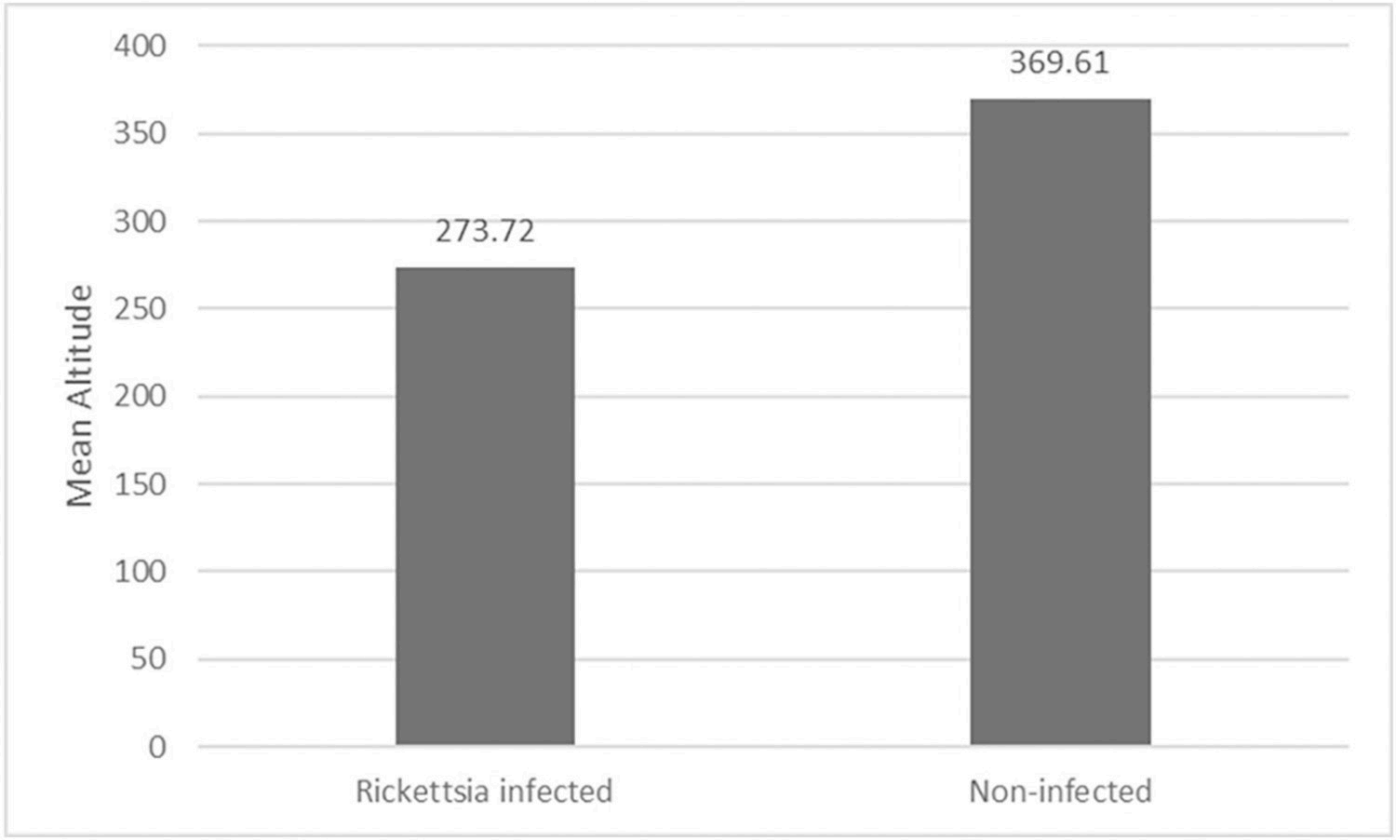
The relationship between the mean altitude of *Rickettsia* positive (n = 46) and negative (n = 300) *I*. *ovatus*. Welch t-tests revealed a significant difference in the mean altitude between *Rickettsia-infected* and non-infected *I*. *ovatus* at *p* < 0.05. Populations with combined sites are labeled A to H.

**Fig 4:**
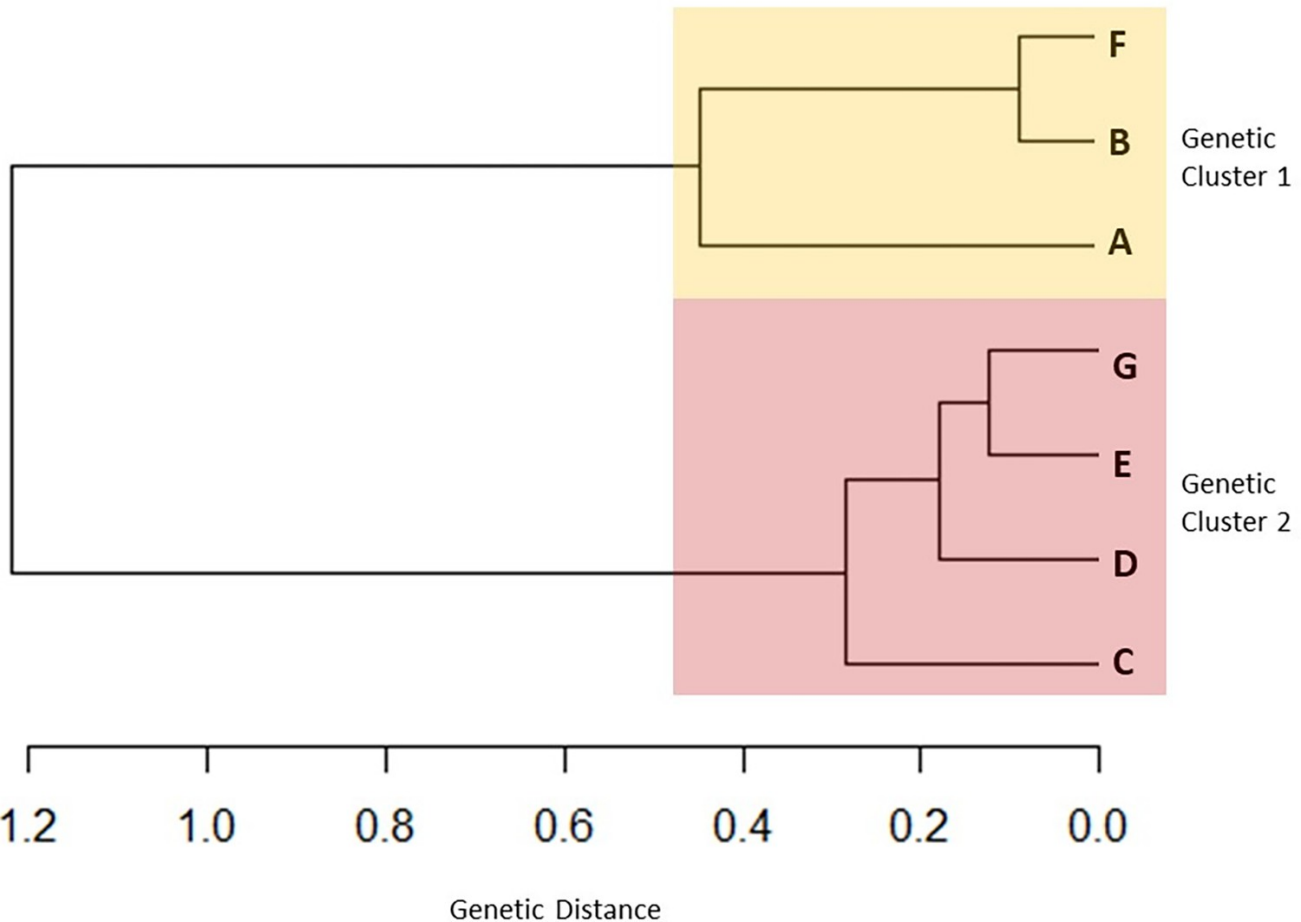
An unweighted pair group method with the arithmetic mean (UPGMA) dendrogram of *I*. *ovatus* based on the pairwise genetic distance (*F*_ST_) of *cox1* among the 7 populations across Niigata Prefecture, Japan. We excluded one population due to the limited number of samples.

The *cox1* haplotype network of *I*. *ovatus* (Fig 1) revealed four genetic groups, wherein three genetic groups (1, 2, and 3) were distributed along different altitudinal gradients, as shown in Fig 2. These four genetic groups were concordant with the four clusters found in the *I*. *ovatus* phylogenetic tree (S1 Fig). The habitat distribution of genetic group 3 was limited to high altitude sites only (range = 16–912 m.a.s.l.), whereas genetic groups 1 (255–471 m.a.s.l.) and 2 (84–1354 m.a.s.l.) were distributed at lower altitudes (Fig 2). The Welch t-test revealed a significant difference between the mean altitudes of genetic groups 1 and 3 at *p* < 0.05 (Table 2); however, no significant difference was observed between genetic groups 1 and 2 or groups 2 and 3. We found a significant difference in the *Rickettsia* infection rates between *I*. *ovatus* genetic groups 1 and 2 based on the z-score test, but no significant difference between groups 1 and 3 or groups 2 and 3 (Table 2). The mean altitude between the *Rickettsia-*infected (= 273.72 m.a.s.l.) and non-infected *I*. *ovatus* (= 369.61 m.a.s.l.) revealed a significant difference based on the Welch t-test at *p* < 0.05 (Fig 3). The UPGMA dendrogram of *I*. *ovatus* revealed two genetic clusters, 1 and 2 using the genetic distance among the seven populations excluding one population due to the limited number of samples (Fig 4).

**Table 2:** The differences in *Rickettsia* infection rates and mean altitude in *I*. *ovatus* haplotype groups and cluster dendrogram groups. The table shows the distribution of *Rickettsia*-infected and uninfected *I*. *ovatus* and the mean altitude in each of the haplotype groups as shown in Fig 1 *(I*. *ovatus* haplotype network) and the cluster dendrogram in Fig 4 (*I*. *ovatus* cluster dendrogram). The z-score test showed a significant difference at *p* < 0.05 between the *Rickettsia* detection rates in haplotype groups 1 and 2 (indicated by ^ab^) and in cluster dendrogram groups 1 and 2. The Welch t-test at *p* < 0.05 revealed a significant difference in the mean altitude of haplotype groups 1 and 3 and cluster dendrogram groups 1 and 2 indicated by ^ab^. Haplotype group 4 was not included in the analysis due to its low sample size.

Haplotype group	*Rickettsia* infection rate			Mean altitude
	Positive	Negative	Detection rate	
**1**	1	92	1.07%^ab^	294^ab^
**2**	28	99	22.04%^ab^	341^a^
**3**	16	110	12.70% ^a^	374^ab^
**Dendrogram Group**				
**1**	4	120	3.23% ^ab^	30
**2**	40	227	14.98% ^ab^	345

The cox1 haplotype network of *H*. *flava* displayed two genetic groups (S2 Fig) consistent with the *H*. *flava* phylogenetic tree (S3 Fig). No significant difference between the *Rickettsia* infection rates of *H*. *flava* genetic groups 1 and 2 was observed using the z-score test at *p* < 0.05 (S3 Table).

## Discussion

Our findings support our hypothesis that a genetically structured tick population, such as *I*. *ovatus* is associated with the *Rickettsia* infection rate to be spatially heterogenous due to limited gene flow along an altitudinal gradient. Our results were consistent with our previous study [11] which suggested that the low mobility of the host species for immature *I*. *ovatus* contributed to low gene flow in the tick populations. Despite the addition of new samples of *I*. *ovatus* and *H*. *flava*, we found a similar pattern of population genetic structure from the previous study of [11] thus supporting the robustness of their population genetic structure results. The low *I*. *ovatus* gene flow along the altitudinal gradient might have caused the spatial heterogeneity of *Rickettsia* infection rates among these populations, which is supported by the significant difference found in *Rickettsia* infection rates between genetic clusters 1 and 2. A similar pattern was observed in the studies of [49–51] which found that the infection rate of *Borrelia burgdorferi*, the causative agent of Lyme disease, decreased in ticks along the altitudinal gradient. Low gene flow can cause infected and uninfected ticks to have limited opportunities to traverse a wider spatial area thus causing a heterogeneous *Rickettsia* infection rate [52, 53].

We found two genetic groups in the *H*. *flava* haplotype network, but no significant difference in the *Rickettsia* infection rates between the two groups. These results might be due to the high gene flow observed in the *H*. *flava* populations, which enable *Rickettsia*-infected and uninfected *H*. *flava* individuals to traverse between the study sites. The high mobility of the large mammalian hosts used by adult *H*. *flava* and avian-mediated dispersal during their immature stage probably contributed to their homogenized population genetic structure [11], and the resulting homogenized *Rickettsia* infection rates. Large mammalian hosts and birds have a wide dispersal range that enables the broader movement of *Rickettsia*-infected ticks, as observed in previous studies of *Amblyomma Americanum* [13, 14, 54], *H*. *flava* [11], and *I*. *ricinus* [55]. Birds are especially good at dispersing over large areas since they can easily traverse landscape barriers such as mountains, fences, glaciers, and oceans that would be difficult for mammals to cross [56].

The different *Rickettsia* infection rates and altitudinal ranges between the *I*. *ovatus* phylogenetic groups maybe caused by diverse factors such as host availability and distribution, other environmental factors such as climate and vegetation, and anthropogenic factors such as urbanization. However, the adaptive evolutionary theory, which states that organisms adjust to new or severe changes in their environment to become better suited to their habitat [57, 58] maybe the best explanation for our results. Based on the relationship between the *I*. *ovatus* phylogenetic groups and their mean attitudes, *I*. *ovatus* might be undergoing local adaptation along the altitudinal gradient due to the higher genetic differentiation between populations as supported by the significant global *F*_ST_ (0.4154) found in *I*. *ovatus*. Based on isolation by environment (IBE), genetic differentiation will increase with increased environmental differences independent of geographic distances [33, 34, 59]. Thus in our study, the addition of *I*. *ovatus* together with the published data of [11] collected from an altitudinal gradient have shown genetic differences and different *Rickettsia* infection rate. When environmental conditions differ, the success of immigration in a new habitat is reduced, which may increase the genetic fixation rate due to a lower chance of outcrossing; thereby enhancing genetic isolation [60]. Thus, the lower gene flow along the altitudinal gradient reduced the spatial homogeneity of *Rickettsia* infection rates among the *I*. *ovatus* tick populations, thus causing the different *Rickettsia* infection rates obtained.

The occurrence of local adaptation in tick populations could affect the future of the tick-borne disease landscape. Environmental factors, such as precipitation, temperature, and altitude, have been shown to drive population differentiation in insects, such as *Anopheles* mosquitoes and *Drosophila* flies [61–63], but studies on the environmental adaptation of ixodid ticks, such as *I*. *ovatus*, and its *Rickettsia* infection susceptibility, have not yet been performed. [7] suggested that environmental conditions that affect bird hosts can also affect the local adaptation of ticks. Few studies have assessed such local adaptation in multiple organisms with varying dispersal abilities [64–68] and is an area in need of future research.

One of the limitations of this study is the use of one mitochondrial gene *cox1* which limited us to compare our results with other target genes to highly support our findings. If markers with high mutation rates or many markers were used, it might have been possible to look at even finer population genetic structure and see differences in infection rates among the subdivided populations. Despite this, we were able to determine the relationship between the tick population genetic structure and *Rickettsia* infection rates as influenced by the altitudinal gradient. The mitochondrial *cox1* gene has been widely used for population genetic analysis of many tick species and was proven to be informative in determining the relationship from the subfamily to the population levels [69–73]. Mitochondrial genes have a mutation rate that is useful in species-level phylogenetics and can be used for wide geographic ranges however its resolution is not fine enough to study species selection [10]. In future studies, we suggest including additional mitochondrial genes and or nuclear genes.

Since ticks are blood-sucking ectoparasites, they directly influence their mammalian hosts and the pathogens they transmit [74–76]. The interaction between the vector (tick), host, and pathogen (*Rickettsia*) is essential in understanding and predicting the risk and transmission of tick-borne diseases [77]. Understanding the genetic structure of ticks can serve as an alternative indicator to infer the potential spread of its pathogen [78]. Our study found relationships between (1) the population genetic structure of ticks and the corresponding *Rickettsia* infection rates, (2) altitude and the population genetic structure of ticks, and (3) altitude and *Rickettsia* infection rates. Though our results can provide a useful information about the tick distribution and possible potential spread of pathogens, there are some factors that should also be considered to apply our results such as ticks can have different mammalian hosts during different life stages in the field that have varying hosts mobility and other environmental factors can also affect such as temperature, humidity etc.. can also be a factor. We found that host mobility may influence the genetic structure of ixodid ticks. This information can be used to design more effective tick-borne disease control programs that focus on screening and detecting pathogens found in ticks and their mammalian hosts. For example, patterns of disease transmission from ticks with a high genetic divergence and less mobile hosts, such as *I*. *ovatus*, are likely due to the movement of infected hosts rather than infected ticks. Thus, screening prospective tick hosts for *Rickettsia* infection would be more suitable in this example. We suggest screening the hosts of immature *I*. *ovatus*, such as small rodents, instead of screening ticks. The *Rickettsia* infection rate in tick genetic groups can predict the spread of tick-borne diseases caused by *Rickettsia*, such as the Japanese spotted fever. We also found that altitude may influence the *Rickettsia* infection rate of *I*. *ovatus* genetic groups. This information can be used to determine the high-risk areas (e.g., lowland, mountains, etc.) of tick-borne diseases along an altitudinal gradient. Genetically structured arthropod vectors, such as ticks, can have different vector competencies, and environmental factors, such as altitudinal gradients, that can influence the vector’s ability to acquire, transmit, and maintain the pathogen infection [79].

## Supporting information

S1 Table Summary of *Ixodes ovatus* and *Haemaphysalis flava* collected from the different locations of Niigata Prefecture and its corresponding sample number and number of *Rickettsia* infection per site. (XLSX)

S2 Table Summary of PCR primers used in the detection of Spotted fever group *Rickettsia*. (DOCX)

S3 Table The difference in *Rickettsia* infection rates in *H*. *flava* haplotype groups and a table showing the distribution of *Rickettsia*-infected and uninfected *H*. *flava* in each genetic groups as shown in S2 Fig *(H*. *flava* median-joining network). The results of the z-score test for two populations proportions at *p* < 0.05 showed no significant difference between the *Rickettsia* detection rate in haplotype groups 1 and 2. The Welch t-test at *p* < 0.05 revealed no significant difference in the mean altitude of the two groups.
(XLSX)

S1 Fig Phylogenetic tree from the BEAST analysis of 59 haplotype *cox1* sequences of *I*. *ovatus*. The blue-labeled haplotypes indicate the presence of *Rickettsia* infection. The red parentheses provide the number of *Rickettsia*-positive individuals per haplotype. The black labeled haplotypes are negative for *Rickettsia* infection.
(DOCX)

S2 Fig Median-joining network of the 66 *cox1* haplotype sequences of *Rickettsia* positive and negative *H*. *flava*. Haplotype groups are indicated as 1 and 2.
(DOCX)

S3 Fig Phylogenetic tree from the BEAST analysis of 66 haplotype *cox1* sequences of *H*. *flava*. The blue-labeled haplotypes indicate *Rickettsia* infection in individual samples. The parentheses in red provide the number of *Rickettsia-*infected ticks. The black-labeled haplotypes are negative for *Rickettsia* infection.
(DOCX)

## References

[1] Neumann D. Eine hysterische Hausepidemie. Monatsschrift für Psychiatrie und Neurologie. 1899 Jun 1;5(6):405–10.

[2] Miyamoto K., Nakao M., Fujita H., Sato F., 1993. The ixodid ticks on migratory birds in Japan and the isolation of Lyme disease spirochetes from bird-feeding ticks. Med. Entomol. and zool., 44(4), 315–326. doi: 10.7601/mez.44.315

[3] Neumann H. Zur Behandlung der Pleuritis exsudativa. DMW-Deutsche Medizinische Wochenschrift. 1897 Jan;23(05):73–5.

[4] Yu XJ, Liang MF, Zhang SY, Liu Y, Li JD, Sun YL, et al. Fever with thrombocytopenia associated with a novel bunyavirus in China. N. Engl. J. Med. 2011 Apr 21;364(16):1523–32. doi: 10.1056/NEJMoa1010095 21410387 PMC3113718

[5] Yamaji K, Aonuma H, Kanuka H. Distribution of tick-borne diseases in Japan: Past patterns and implications for the future. J. Infect. Chemother.2018 Jul 1;24(7):499–504. doi: 10.1016/j.jiac.2018.03.012 29685854

[6] Arai R, Sato M, Kato M, Aoki J, Nishida A, Watanabe K, et al. Spotted fever group rickettsiae (SFGR) detection in ticks following reported human case of Japanese spotted fever in Niigata Prefecture, Japan. Sci. Rep. 2021 Jan 28;11(1):2595. doi: 10.1038/s41598-021-81587-9 33510192 PMC7844008

[7] McCoy KD, Boulinier T, Tirard C, Michalakis Y. Host specificity of a generalist parasite: genetic evidence of sympatric host races in the seabird tick *Ixodes uriae*. J. Evol. Biol. 2001 May 9;14(3):395–405. doi: 10.1046/j.1420-9101.2001.00290.x

[8] Oliver JD, Bennett SW, Beati L, Bartholomay LC. Range expansion and increasing *Borrelia burgdorferi* infection of the tick *Ixodes scapularis* (Acari: Ixodidae) in Iowa, 1990–2013. J. Med. Entomol. 2017 Nov 7;54(6):1727–34. doi: 10.1093/jme/tjx121 28633503

[9] Agany DD, Pietri JE, Gnimpieba EZ. Assessment of vector-host-pathogen relationships using data mining and machine learning. Comput. Struct. Biotechnol. J. 2020 Jan 1;18:1704–21.doi: 10.1016/j.csbj.2020.06.031 32670510 PMC7340972

[10] Araya-Anchetta A, Busch JD, Scoles GA, Wagner DM. Thirty years of tick population genetics: a comprehensive review. Infect. Genet. Evol. 2015 Jan 1;29:164–79. doi: 10.1016/j.meegid.2014.11.008 25461844

[11] Regilme MA, Sato M, Tamura T, Arai R, Sato MO, Ikeda S, et al. Comparative population genetic structure of two ixodid tick species (Acari: Ixodidae)(*Ixodes ovatus* and *Haemaphysalis flava*) in Niigata prefecture, Japan. Infect. Genet. Evol. 2021 Oct 1;94:104999. doi: 10.1016/j.meegid.2021.104999 34256167

[12] Estrada-Peña AJ. Ticks as vectors: taxonomy, biology and ecology. Rev. Sci. Tech. 2015 Apr 1;34(1):53–65. doi: 10.20506/rst.34.1.2345 26470449

[13] Mixson TR, Lydy SL, Dasch GA, Real LA. Inferring the population structure and demographic history of the tick, *Amblyomma americanum* Linnaeus. J. Vector Ecol. 2006 Jun;31(1):181–92. 10.3376/1081-1710(2006)31[181:itpsad]2.0.co; 2. 16859107

[14] Trout RT, Steelman CD, Szalanski AL. Population genetics of *Amblyomma americanum* (Acari: Ixodidae) collected from Arkansas. J. Med. Entomol. 2014 Dec 1;47(2):152–61. doi: 10.1603/me09106 20380295

[15] Guglielmone AA, Nava S, Mastropaolo M, Mangold AJ. Distribution and genetic variation of *Amblyomma triste* (Acari: Ixodidae) in Argentina. Ticks Tick Borne Dis. 2013 Sep 1;4(5):386–90. doi: 10.1016/j.ttbdis.2013.01.009 23647652

[16] Lampo M, Rangel Y, Matat A. Population genetic structure of a three-host population genetic in *Amblyommma dissimile*. J. Parasitol. 2015;84:1137–42. doi: 10.2307/3284662 9920303

[17] Dougherty ER, Seidel DP, Carlson CJ, Spiegel O, Getz WM. Going through the motions: incorporating movement analyses into disease research. Ecol. Lett. 2018 Apr;21(4):588–604. doi: 10.1111/ele.12917 29446237

[18] Regoes RR, Nowak MA, Bonhoeffer S. Evolution of virulence in a heterogeneous host population. Evol. 2000 Feb;54(1):64–71. doi: 10.1111/j.0014-3820.2000.tb00008.x 10937184

[19] Boots M, Mealor M. Local interactions select for lower pathogen infectivity. Sci. 2007 Mar 2;315(5816):1284–6.doi: 10.1126/science.1137126 17332415

[20] Lion S, Gandon S. Evolution of spatially structured host–parasite interactions. Journal of Evol. Biol. 2015 Jan;28(1):10–28. doi: 10.1111/jeb.12551 25439133

[21] Chin KM, Wolfe MS. Selection on *Erysiphe graminis* in pure and mixed stands of barley. Plant Pathol. 1984;33:535–546. doi: 10.1111/j.1365-3059.1984.tb02878.x

[22] Thrall PH, Burdon JJ. Evolution of virulence in a plant host-pathogen metapopulation. Science. 2003;299:1735–1737. doi: 10.1126/science.1080070 12637745

[23] White PS, Choi A, Pandey R, Menezes A, Penley M, Gibson AK, et al. Host heterogeneity mitigates virulence evolution. Biol Lett. 2020;16:20190744. doi: 10.1098/rsbl.2019.0744 31992149 PMC7013476

[24] Gibson AK, Baffoe‐Bonnie H, Penley MJ, Lin J, Owens R, Khalid A, et al. The evolution of parasite host range in heterogeneous host populations. J Evol Biol. 2020;33:773–782. doi: 10.1111/jeb.13608 32086852 PMC7275899

[25] Bono LM, Gensel CL, Pfennig DW, Burch CL. Competition and the origins of novelty: experimental evolution of niche-width expansion in a virus. Biol Lett. 2013;9:20120616. doi: 10.1098/rsbl.2012.0616 23075527 PMC3565482

[26] Streicker DG, Winternitz JC, Satterfield DA, Condori-Condori RE, Broos A, Tello C, et al. Host–pathogen evolutionary signatures reveal dynamics and future invasions of vampire bat rabies. Proc Natl Acad Sci U S A. 2016;113:10926–10931. doi: 10.1073/pnas.1606587113 27621441 PMC5047211

[27] Fountain‐Jones NM, Craft ME, Funk WC, Kozakiewicz C, Trumbo DR, Boydston EE, et al. Urban landscapes can change virus gene flow and evolution in a fragmentation‐sensitive carnivore. Mol Ecol. 2017;26:6487–6498. doi: 10.1111/mec.14375 28987024

[28] Daniel M, Malý M, Danielová V, Kříž B, Nuttall P. Abiotic predictors and annual seasonal dynamics of *Ixodes ricinus*, the major disease vector of Central Europe. Parasit Vectors. 2015;8:1–12. doi: 10.1186/s13071-015-1092-y 26385470 PMC4575455

[29] Eisen RJ, Eisen L, Beard CB. County-scale distribution of *Ixodes scapularis* and *Ixodes pacificus* (Acari: Ixodidae) in the continental United States. J Med Entomol. 2016;53:349–386. doi: 10.1093/jme/tjv237 26783367 PMC4844559

[30] Chapman MA, Hiscock SJ, Filatov DA. Genomic divergence during speciation driven by adaptation to altitude. Mol Biol Evol. 2013;30:2553–2567. doi: 10.1093/molbev/mst168 24077768 PMC3840311

[31] Osborne OG, Batstone TE, Hiscock SJ, Filatov DA. Rapid speciation with gene flow following the formation of Mt. Etna. Genome Biol Evol. 2013;5:1704–1715. doi: 10.1093/gbe/evt127 23973865 PMC3787679

[32] Polato NR, Gray MM, Gill BA, Becker CG, Casner KL, Flecker AS, et al. Genetic diversity and gene flow decline with elevation in montane mayflies. Heredity. 2017 Aug;119(2):107–116. doi: 10.1038/hdy.2017.23 28489073 PMC5520546

[33] Wang J., Summers K., 2010. Genetic structure is correlated with phenotypic divergence rather than geographic isolation in the highly polymorphic strawberry poison-dart frog. Mol. Ecol., 19, 447–458. doi: 10.1111/j.1365-294X.2009.04465.x 20025652

[34] Bradburd G.S., Ralph P.L., Coop G.M., 2013. Disentangling the effects of geographic and ecological isolation on genetic differentiation. Evolution, 67, 3258–3273. doi: 10.1111/evo.12193 24102455 PMC3808528

[35] Sexton J.P., Hangartner S.B., Hoffmann A.A., 2014. Genetic isolation by environment or distance: which pattern of gene flow is most common?. Evol., 68, 1–15. doi: 10.1111/evo.12258 24111567

[36] Yamaguti N., Tipton V. J., Keegan H. L., & Toshioka S. (1971). Ticks of Japan, Korea, and the Ryukyu islands. Brigham Young University Science Bulletin, Biological Series, 15, 1.

[37] Ishikura M. et al. Phylogenetic analysis of spotted fever group rickettsiae based on gltA, 17-kDa, and rOmpA genes amplified by nested PCR from ticks in Japan. Microbiol. Immunol. 47, 823–832 (2003). doi: 10.1111/j.1348-0421.2003.tb03448.x 14638993

[38] Regnery R. L., Spruill C. L. & Plikaytis B. D. Genotypic identification of rickettsiae and estimation of intraspecies sequence divergence for portions of two rickettsial genes. J. Bacteriol. 173, 1576–1589 (1991). doi: 10.1128/jb.173.5.1576-1589.1991 1671856 PMC207306

[39] Fournier P.-E., Roux V. & Raoult D. Phylogenetic analysis of spotted fever group rickettsiae by study of the outer surface protein rOmpA. Int. J. Syst. Bacteriol. 48, 839–849 (1998). doi: 10.1099/00207713-48-3-839 9734038

[40] Paddock C. D. et al. Rickettsia parkeri: A newly recognized cause of spotted fever rickettsiosis in the United States. Clin. Infect. Dis. 38, 805–811 (2004). doi: 10.1086/381894 14999622

[41] Choi Y.-J. et al. Spotted fever group and typhus group rickettsioses in humans, South Korea. Emerg. Infect. Dis. 11, 237–244 (2005). doi: 10.3201/eid1102.040603 15752441 PMC3320442

[42] Vrijenhoek R. DNA primers for amplification of mitochondrial cytochrome c oxidase subunit I from diverse metazoan invertebrates. Mol Mar Biol Biotechnol. 1994 Oct 1;3(5):294–9. . 7881515

[43] Maddison W.P., Maddison D.R., 2011. Mesquite: A modular system for evolutionary analysis. Available at: http://mesquiteproject.org.

[44] Rozas J, Ferrer-Mata A, Sánchez-DelBarrio JC, Guirao-Rico S, Librado P, Ramos-Onsins SE, et al. DnaSP 6: DNA sequence polymorphism analysis of large datasets. Mol Biol Evol. 2017 Dec 1;34(12):3299–3302. doi: 10.1093/molbev/msx248 29029172

[45] Excoffier L., & Lischer H. E. L. (2010). Arlequin suite ver 3.5: A new series of programs to perform population genetics analyses under Linux and Windows. Molecular Ecology Resources, 10(3), 564–567. doi: 10.1111/j.1755-0998.2010.02847.x 21565059

[46] Paradis E., Schliep K., 2018. APE 5.0: an environment for modern phylogenetics and evolutionary analyses in R. Bioinformatics, 35, 526–528. doi: 10.1093/bioinformatics/bty633 30016406

[47] Bandelt H.J., Forster P., Röhl A., 1999. Median-joining networks for inferring intraspecific phylogenies. Mol. Biol. Evol. 16, 37–48. doi: 10.1093/oxfordjournals.molbev.a026036 10331250

[48] Drummond AJ, Rambaut A. BEAST: Bayesian evolutionary analysis by sampling trees. BMC Evol Biol. 2007 Nov 8;7:214. doi: 10.1186/1471-2148-7-214 17996036 PMC2247476

[49] Burri C., Cadenas F.M., Douet V., Moret J., Gern L., 2007. *Ixodes ricinus* density and infection prevalence of *Borrelia burgdorferi* sensu lato along a north-facing altitudinal gradient in the Rhône Valley (Switzerland). Vector-Borne and Zoonotic Diseases, 7, 50–58. doi: 10.1089/vbz.2006.0569 17417957

[50] Jouda F., Perret J.L., Gern L., 2004. *Ixodes ricinus* density, and distribution and prevalence of *Borrelia burgdorferi* sensu lato infection along an altitudinal gradient. J. Med. Entomol. 41, 162–169. doi: 10.1603/0022-2585-41.2.162 15061274

[51] Moran-Cadenas F, Schneider H, Lommano E, Burri C, Moret J, Gern L. A comparison of two DNA extraction approaches in the detection of *Borrelia burgdorferi* sensu lato from live *Ixodes ricinus* ticks by PCR and reverse line blotting. Vector Borne Zoonotic Dis. 2007 Dec 1;7(4):555–62. doi: 10.1089/vbz.2006.0596 17979538

[52] Gooding R.H. Genetic variation in arthropod vectors of disease-causing organisms: Obstacles and opportunities. Clin. Microbiol. Rev. 1996; 9: 301.doi: 10.1128/CMR.9.3.301 8809462 PMC172895

[53] Páez-Triana L., Muñoz M., Herrera G., Moreno-Pérez D.A., Tafur-Gómez G.A., Montenegro D., et al., 2021. Genetic diversity and population structure of *Rhipicephalus sanguineus* sensu lato across different regions of Colombia. Parasit Vectors, 14, 1–11. doi: 10.1186/s13071-021-04898-w 34425895 PMC8383428

[54] Reichard M.V., Kocan A.A., Van Den Bussche A., Barker R.W., Wyckoff J., Ewing S.A., 2005. Sequence variation of the ribosomal DNA second internal transcribed spacer region in two spatially distinct populations of *Amblyomma americanum* (L.)(Acari: Ixodidae). J. Parasitol. 91, 260–264. doi: 10.1645/GE-292R1 15986597

[55] Casati S., Bernasconi M.V., Gern L., Piffaretti J.C., 2008. Assessment of intraspecific mtDNA variability of European *Ixodes ricinus* sensu stricto (Acari: Ixodidae). Infect. Genet. Evol. 8, 152–158. doi: 10.1016/j.meegid.2007.11.007 18206426

[56] Hasle G., 2013. Transport of ixodid ticks and tick-borne pathogens by migratory birds. Front. Cell. Infect. Microbiol., 3, 48. doi: 10.3389/fcimb.2013.00048 24058903 PMC3767891

[57] Kawecki TJ, Ebert D. Conceptual issues in local adaptation. Ecol Lett. 2004 Dec;7(12):1225–1241. doi: 10.1111/j.1461-0248.2004.00684.x

[58] Gregory TR. Understanding natural selection: essential concepts and common misconceptions. Evol Educ Outreach. 2009 Jun;2(2):156–175. doi: 10.1007/s12052-009-0128-1

[59] Wang IJ, Bradburd GS. Isolation by environment. Mol. Ecol. 2014 Dec;23(23):5649–5662. doi: 10.1111/mec.12938 25256562

[60] Jiang S, Luo MX, Gao RH, Zhang W, Yang YZ, Li YJ, et al. Isolation-by-environment as a driver of genetic differentiation among populations of the only broad-leaved evergreen shrub *Ammopiptanthus mongolicus* in Asian temperate deserts. Sci Rep. 2019 Jul 30;9(1):1–14. doi: 10.1038/s41598-019-48472-y 31427616 PMC6700151

[61] Cheng C., White B.J., Kamdem C., Mockaitis K., Costantini C., Hahn M.W., et al., 2012. Ecological genomics of *Anopheles gambiae* along a latitudinal cline: a population-resequencing approach. Genetics, 190(4), 1417–1432. doi: 10.1534/genetics.112.139899 22209907 PMC3316653

[62] Kapun M., Barrón M.G., Staubach F., Obbard D.J., Wiberg R.A.W., Vieira J., et al. (2020). Genomic analysis of European *Drosophila melanogaster* populations reveals longitudinal structure, continent-wide selection, and previously unknown DNA viruses. Mol. Biol. Evol., 37, 2661–2678. doi: 10.1093/molbev/msaa120 32413142 PMC7475034

[63] Love RR, Steele AM, Coulibaly MB, Traore SF, Emrich SJ, Fontaine MC, et al. Chromosomal inversions and ecotypic differentiation in *Anopheles gambiae*: the perspective from whole-genome sequencing. Mol Ecol. 2016 Dec;25(23):5889–906. doi: 10.1111/mec.13888 27759895 PMC5130611

[64] Exposito-Alonso M, Vasseur F, Ding W, Wang G, Burbano HA, Weigel D. Genomic basis and evolutionary potential for extreme drought adaptation in *Arabidopsis thaliana*. Nat Ecol Evol. 2018 Feb;2(2):352–8., doi: 10.1038/s41559-017-0423-0 29255303 PMC5777624

[65] Hancock AM, Brachi B, Faure N, Horton MW, Jarymowycz LB, Sperone FG, et al. Adaptation to climate across the *Arabidopsis thaliana* genome. Science. 2011 Oct 7;334(6052):83–6.doi: 10.1126/science.1209244 21980108

[66] Zhen Y, Harrigan RJ, Ruegg KC, Anderson EC, Ng TC, Lao S, et al. Genomic divergence across ecological gradients in the Central African rainforest songbird (*Andropadus virens*). Mol. Ecol. 2017 Oct;26(19):4966–77.doi: 10.1111/mec.14270 28752944

[67] Ahrens CW, Byrne M, Rymer PD. Standing genomic variation within coding and regulatory regions contributes to the adaptive capacity to climate in a foundation tree species. Mol Ecol. 2019 May;28(10):2502–16. doi: 10.1111/mec.15092 30950536

[68] Miller AD, Hoffmann AA, Tan MH, Young M, Ahrens C, Cocomazzo M, et al. Local and regional scale habitat heterogeneity contribute to genetic adaptation in a commercially important marine mollusc (*Haliotis rubra*) from southeastern Australia. Mol. Ecol. 2019 Jun;28(12):3053–72. doi: 10.1111/mec.15128 31077479

[69] Crosbie PR, Boyce WM, Rodwell TC. DNA sequence variation in *Dermacentor hunteri* and estimated phylogenies of *Dermacentor* spp.(Acari: Ixodidae) in the New World. J. Med. Entomol. 1998 May 1;35(3):277–88. doi: 10.1093/jmedent/35.3.277 9615547

[70] Norris DE, Klompen JS, Keirans JE, Black IV WC. Population genetics of *Ixodes scapularis* (Acari: Ixodidae) based on mitochondrial 16S and 12S genes. J. Med. Entomol.1996 Jan 1;33(1):78–89. doi: 10.1093/jmedent/33.1.78 8906909

[71] Qiu WG, Dykhuizen DE, Acosta MS, Luft BJ. Geographic uniformity of the Lyme disease spirochete (*Borrelia burgdorferi*) and its shared history with tick vector (*Ixodes scapularis*) in the northeastern United States. Genetics. 2002 Mar 1;160(3):833–49. doi: 10.1093/genetics/160.3.833 11901105 PMC1462027

[72] Rich SM, Caporale DA, Telford 3rd SR, Kocher TD, Hartl DL, Spielman A. Distribution of the *Ixodes ricinus*-like ticks of eastern North America. Proceedings of the National Academy of Sciences. 1995 Jul 3;92(14):6284–8. doi: 10.1073/pnas.92.14.6284 7603983 PMC41502

[73] de la Fuente J, Almazán C, Van Den Bussche RA, Bowman J, Yoshioka JH, Kocan KM. Characterization of genetic diversity in *Dermacentor andersoni* (Acari: Ixodidae) with body size and weight polymorphism. Exp. Parasitol. 2005 Jan 1;109(1):16–26. doi: 10.1016/j.exppara.2004.10.004 15639135

[74] Anderson JF, Magnarelli LA. Biology of ticks. Infectious disease clinics of North America. 2008 Jun 1;22(2):195–215. doi: 10.1016/j.idc.2007.12.006 18452797

[75] Hill CA, Wikel SK. The *Ixodes scapularis* Genome Project: an opportunity for advancing tick research. Trends Parasitol. 2005 Apr 1;21(4):151–3.doi: 10.1016/j.pt.2005.02.004 15780833

[76] Van Zee JP, Geraci NS, Guerrero FD, Wikel SK, Stuart JJ, Nene VM, et al. Tick genomics: the *Ixodes* genome project and beyond. Int. J. Parasitol. 2007 Oct 1;37(12):1297–305. doi: 10.1016/j.ijpara.2007.05.011 17624352

[77] Jongejan F, Nene V, de la Fuente J, Pain A, Willadsen P. Advances in the genomics of ticks and tick-borne pathogens. Trends Parasitol. 2007 Sep 1;23(9):391–6. doi: 10.1016/j.pt.2007.07.004 17656151

[78] Gray JS. Biology of Ixodes species ticks in relation to tick-borne zoonoses. Wiener Klinische Wochenschrift. 2002 Jul 1;114(13–14):473–8. 12422585

[79] Parola P, Paddock CD. Travel and tick-borne diseases: Lyme disease and beyond. Travel Med Infect Dis. 2018;26:1–2. doi: 10.1016/j.tmaid.2018.09.010 30267770

