# Supplementary material for: Genetic structure and Rickettsia infection rates in Ixodes ovatus and Haemaphysalis flava ticks across different altitudes
Source: PLoS One. 2024 Mar 13;19(3):e0298656. doi: 10.1371/journal.pone.0298656 (PMC10936840; doi:10.1371/journal.pone.0298656)
Supplement: S1 Fig — The blue-labeled haplotypes indicate the presence of Rickettsia infection. The red parentheses provide the number of Rickettsia-positive individuals per haplotype. The black labeled haplotypes are negative for Rickettsia infection. (DOCX) [file pone.0298656.s004.docx]

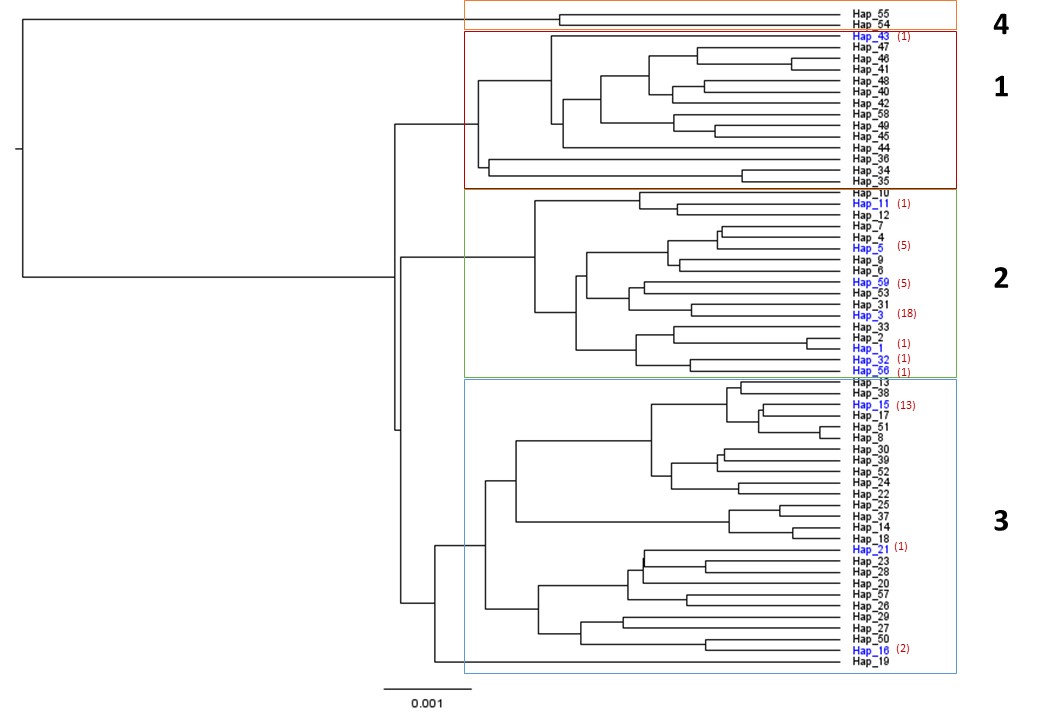


**Supplementary Figure 1.** Phylogenetic tree from BEAST analysis of 59 haplotype *cox1* sequences of *I. ovatus.* The blue labelled haplotypes indicate the presence of *Rickettsia* infection. The red parenthesis beside is the number of *Rickettsia* positive individuals per haplotype. The black labelled haplotypes are negative for *Rickettsia* infection.
