# Supplementary material for: Genetic structure and Rickettsia infection rates in Ixodes ovatus and Haemaphysalis flava ticks across different altitudes
Source: PLoS One. 2024 Mar 13;19(3):e0298656. doi: 10.1371/journal.pone.0298656 (PMC10936840; doi:10.1371/journal.pone.0298656)
Supplement: S2 Fig — Haplotype groups are indicated as 1 and 2. (DOCX) [file pone.0298656.s005.docx]

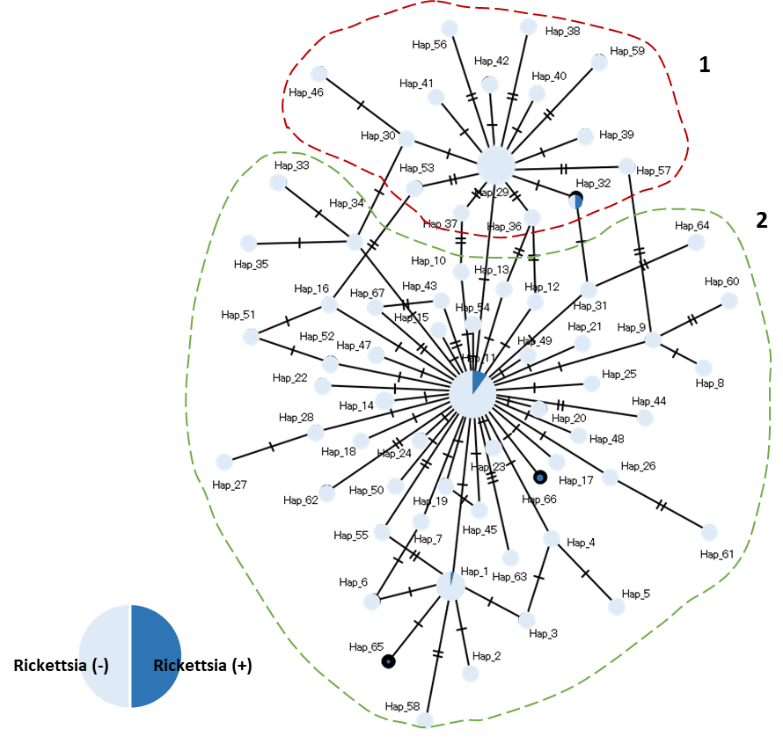


**Supplementary Figure 2.** Median joining network of the 66 *cox1* haplotype sequences of *Rickettsia* positive and negative *H. flava.* Haplotype groups are indicated as 1 and 2.
