# Supplementary material for: Genetic structure and Rickettsia infection rates in Ixodes ovatus and Haemaphysalis flava ticks across different altitudes
Source: PLoS One. 2024 Mar 13;19(3):e0298656. doi: 10.1371/journal.pone.0298656 (PMC10936840; doi:10.1371/journal.pone.0298656)
Supplement: S3 Fig — The blue-labeled haplotypes indicate Rickettsia infection in individual samples. The parentheses in red provide the number of Rickettsia-infected ticks. The black-labeled haplotypes are negative for Rickettsia infection. (DOCX) [file pone.0298656.s006.docx]

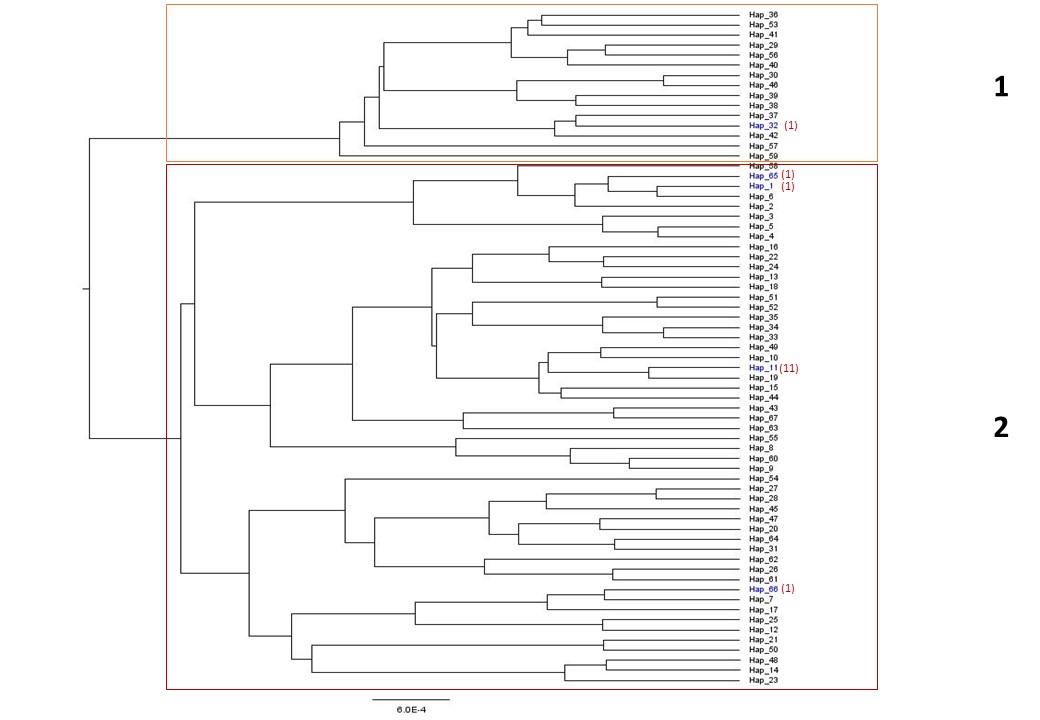


**Supplementary Figure 3.** Phylogenetic tree from BEAST analysis of 66 haplotype *cox1* sequences of *H. flava.* The blue labelled haplotypes indicate *Rickettsia* infection in individual samples. The parenthesis in red shows the number of *Rickettsia* infected tick. The black labelled haplotypes are negative for *Rickettsia* infection.
