# Supplementary material for: Genetic structure and Rickettsia infection rates in Ixodes ovatus and Haemaphysalis flava ticks across different altitudes
Source: PLoS One. 2024 Mar 13;19(3):e0298656. doi: 10.1371/journal.pone.0298656 (PMC10936840; doi:10.1371/journal.pone.0298656)
Supplement: Attachment — Submitted filename: PONE-D-23-12494_reviewer.pdf [file pone.0298656.s007.pdf]

PLOS ONE

Rickettsia infection rate along an altitudinal gradient as influenced by population genetic structure of Ixodid ticks

--Manuscript Draft--

**Manuscript Number:**	PONE-D-23-12494
**Article Type:**	Research Article
**Full Title:**	Rickettsia infection rate along an altitudinal gradient as influenced by population genetic structure of Ixodid ticks
**Short Title:**	Tick population genetic structure and Rickettsia infection rate along an altitudinal gradient
**Corresponding Author:**	Kozo Watanabe<br>Ehime University<br>Matsuyama, JAPAN
**Keywords:**	environment; haplotype; hosts; pathogen; dispersal; vector
**Abstract:**	Ixodid ticks, such as Ixodes ovatus and Haemaphysalis flava, are important vectors of tick-borne diseases in Japan, such as Japanese spotted fever caused by Rickettsia japonica. This study describes the population genetic structure and gene flow of I. ovatus and H. flava and their Rickettsia infection rates along an altitudinal gradient. A total of 346 adult I. ovatus and 243 H. flava were analyzed for the presence of Rickettsia by nested PCR targeting the 17kDA, gltA, rOmpA, and rOmpB genes. Population genetic structure was performed using the mitochondrial cytochrome oxidase 1 (cox1) marker. The Rickettsia infection rates were 13.26% in I. ovatus and 6.17% in H. flava. For I. ovatus, the global FST value revealed significant genetic differentiation among the different populations, whereas H. flava showed non-significant genetic differentiation. The cox1 I. ovatus cluster dendrogram showed two cluster groups, while the haplotype network and phylogenetic tree showed three genetic groups. The two cluster groups and the three genetic groups found in I. ovatus showed a significant difference in their Rickettsia infection rates and mean altitude per group. No significant differences were found in the mean altitude or Rickettsia infection rates of H. flava. Our results suggest that low gene flow in the I. ovatus populations has caused spatially heterogenous Rickettsia infection rates along the altitudinal gradient. This information can be used in understanding the relationship between the tick vector, its pathogen, and environmental factors, such as altitude, and for the control of tick-borne diseases in Japan.
**Order of Authors:**	Maria Angenica Fulo Regilme
	Megumi Sato
	Tsutomu Tamura
	Reiko Arai
	Marcello Otake Sato
	Sumire Ikeda
	Kozo Watanabe
**Additional Information:**	
**Question**	**Response**
**Financial Disclosure**<br><br>Enter a financial disclosure statement that describes the sources of funding for the work included in this submission. Review the submission guidelines for detailed requirements. View published research	This study was supported by the Ministry of Education, Culture, Sports, Science and Technology, Japan (MEXT) to a project on Joint Usage/Research Center– Leading Academia in Marine and Environment Pollution Research (LaMer).The funders had no role in study design, data collection and analysis, decision to publish, or preparation of the manuscript.

Powered by Editorial Manager® and ProduXion Manager® from Aries Systems Corporation

articles from *PLOS ONE* for specific examples.<br><br>This statement is required for submission and **will appear in the published article** if the submission is accepted. Please make sure it is accurate.<br><br>**Unfunded studies**<br>Enter: *The author(s) received no specific funding for this work.*<br><br>**Funded studies**<br>Enter a statement with the following details:<br>• Initials of the authors who received each award<br>• Grant numbers awarded to each author<br>• The full name of each funder<br>• URL of each funder website<br>• Did the sponsors or funders play any role in the study design, data collection and analysis, decision to publish, or preparation of the manuscript?<br>• **NO** - Include this sentence at the end of your statement: *The funders had no role in study design, data collection and analysis, decision to publish, or preparation of the manuscript.*<br>• **YES** - Specify the role(s) played.<br><br>* typeset	
**Competing Interests**<br><br>Use the instructions below to enter a competing interest statement for this submission. On behalf of all authors, disclose any competing interests that could be perceived to bias this work—acknowledging all financial support and any other relevant financial or non-financial competing interests.<br><br>This statement is required for submission and **will appear in the published article** if the submission is accepted. Please make sure it is accurate and that any funding sources listed in your Funding Information later in the submission form are also declared in your Financial Disclosure statement.	The authors have declared that no competing interests exist.

View published research articles from *PLOS ONE* for specific examples.<br><br>**NO authors have competing interests**<br><br>Enter: *The authors have declared that no competing interests exist.*<br><br>**Authors with competing interests**<br><br>Enter competing interest details beginning with this statement:<br><br>*I have read the journal's policy and the authors of this manuscript have the following competing interests: [insert competing interests here]*<br><br>* typeset	
**Ethics Statement**<br><br>Enter an ethics statement for this submission. This statement is required if the study involved:<br><br>• Human participants<br>• Human specimens or tissue<br>• Vertebrate animals or cephalopods<br>• Vertebrate embryos or tissues<br>• Field research<br><br>Write "N/A" if the submission does not require an ethics statement.<br><br>General guidance is provided below. Consult the submission guidelines for detailed instructions. **Make sure that all information entered here is included in the Methods section of the manuscript.**	N/A

**Format for specific study types**<br><br>**Human Subject Research (involving human participants and/or tissue)**<br>• Give the name of the institutional review board or ethics committee that approved the study<br>• Include the approval number and/or a statement indicating approval of this research<br>• Indicate the form of consent obtained (written/oral) or the reason that consent was not obtained (e.g. the data were analyzed anonymously)<br><br>**Animal Research (involving vertebrate animals, embryos or tissues)**<br>• Provide the name of the Institutional Animal Care and Use Committee (IACUC) or other relevant ethics board that reviewed the study protocol, and indicate whether they approved this research or granted a formal waiver of ethical approval<br>• Include an approval number if one was obtained<br>• If the study involved *non-human primates*, add *additional details* about animal welfare and steps taken to ameliorate suffering<br>• If anesthesia, euthanasia, or any kind of animal sacrifice is part of the study, include briefly which substances and/or methods were applied<br><br>**Field Research**<br><br>Include the following details if this study involves the collection of plant, animal, or other materials from a natural setting:<br>• Field permit number<br>• Name of the institution or relevant body that granted permission	
**Data Availability**<br><br>Authors are required to make all data underlying the findings described fully available, without restriction, and from the time of publication. PLOS allows rare exceptions to address legal and ethical concerns. See the PLOS Data Policy and FAQ for detailed information.	Yes - all data are fully available without restriction

A Data Availability Statement describing where the data can be found is required at submission. Your answers to this question constitute the Data Availability Statement and **will be published in the article**, if accepted.<br><br>**Important:** Stating 'data available on request from the author' is not sufficient. If your data are only available upon request, select 'No' for the first question and explain your exceptional situation in the text box.<br><br>Do the authors confirm that all data underlying the findings described in their manuscript are fully available without restriction?	
**Describe where the data may be found in full sentences. If you are copying our sample text, replace any instances of XXX with the appropriate details.**<br><br>• If the data are **held or will be held in a public repository**, include URLs, accession numbers or DOIs. If this information will only be available after acceptance, indicate this by ticking the box below. For example: *All XXX files are available from the XXX database (accession number(s) XXX, XXX.*).<br>• If the data are all contained **within the manuscript and/or Supporting Information files**, enter the following: *All relevant data are within the manuscript and its Supporting Information files.*<br>• If neither of these applies but you are able to provide **details of access elsewhere**, with or without limitations, please do so. For example:<br><br>*Data cannot be shared publicly because of [XXX]. Data are available from the XXX Institutional Data Access / Ethics Committee (contact via XXX) for researchers who meet the criteria for access to confidential data.*<br><br>*The data underlying the results presented in the study are available from (include the name of the third party*	All data generated or analyzed during the study are included in this published article and its supplementary additional files. All the newly generated sequences are available in the Genbank database under the accession numbers _______.Under submission

and contact information or URL).<br>• This text is appropriate if the data are owned by a third party and authors do not have permission to share the data.<br><br>* typeset	
Additional data availability information:	Tick here if the URLs/accession numbers/DOIs will be available only after acceptance of the manuscript for publication so that we can ensure their inclusion before publication.

Manuscript

Click here to access/download;Manuscript;Rev_For PlosOne_MS_Tick Pop Gen Rickettsia_20230322.docx

Full Title: *Rickettsia* infection rate along an altitudinal gradient as influenced by population genetic structure of Ixodid ticks

Short Title: Tick population genetic structure and Rickettsia infection rate along an altitudinal gradient

Author names and affiliations

Maria Angenica F. Regilme[a], Megumi Sato[b], Tsutomu Tamura[c], Reiko Arai[c], Marcello Otake Sato[d], Sumire Ikeda[e], Kozo Watanabe[a]*

[a] Center for Marine Environmental Studies, Ehime University, Matsuyama, Ehime 790-8577, Japan

[b] Graduate School of Health Sciences, Niigata University, Niigata, 951-8518, Japan

[c] Niigata Prefectural Institute of Public Health and Environmental Sciences, Niigata, 950-2144, Japan

[d] Department of Tropical Medicine and Parasitology, Dokkyo Medical University, 880 Kitakobayashi, Mibu-machi, Shimotsuga-gun, Tochigi 321-0293, Japan

[e] Research Laboratories, Research and Development Headquarters, Earth Corporation, Hyogo 678-0192, Japan

Corresponding author*

watanabe.kozo.mj@ehime-u.ac.jp

Center for Marine Environmental Studies, Ehime University, Bunkyo-cho 3, Matsuyama, Ehime, Japan 790-8577

Abstract

Ixodid ticks, such as *Ixodes ovatus* and *Haemaphysalis flava,* are important vectors of tick-borne diseases in Japan, such as Japanese spotted fever caused by *Rickettsia japonica*. This study describes the population genetic structure and gene flow of *I. ovatus* and *H. flava* and their *Rickettsia* infection rates along an altitudinal gradient. A total of 346 adult *I. ovatus* and 243 *H. flava* were analyzed for the presence of *Rickettsia* by nested PCR targeting the 17kDA, *gltA, rOmpA,* and *rOmpB* genes. Population genetic structure was performed using the mitochondrial cytochrome oxidase 1 (*cox1*) marker. The *Rickettsia* infection rates were 13.26% in *I. ovatus* and 6.17% in *H. flava.* For *I. ovatus,* the global F_{ST} value revealed significant genetic differentiation among the different populations, whereas *H. flava* showed non-significant genetic differentiation. The *cox1 I. ovatus* cluster dendrogram showed two cluster groups, while the haplotype network and phylogenetic tree showed three genetic groups. The two cluster groups and the three genetic groups found in *I. ovatus* showed a significant difference in their *Rickettsia* infection rates and mean altitude per group. No significant differences were found in the mean altitude or *Rickettsia* infection rates of *H. flava*. Our results suggest that low gene flow in the *I. ovatus* populations has caused spatially heterogenous *Rickettsia* infection rates along the altitudinal gradient. This information can be used in understanding the relationship between the tick vector, its pathogen, and environmental factors, such as altitude, and for the control of tick-borne diseases in Japan.

Keywords: environment, haplotype, hosts, pathogen, dispersal, vector

1. Introduction

Tick-borne diseases are a significant public health concern in Japan and are transmitted by a diverse range of tick species, such as *Ixodes ovatus* [1] that potentially transmit *Borrelia sp.* causing *Lyme* disease [2] and *Haemaphysalis flava* [3], which transmits *Rickettsia japonica* and is a suspected vector of severe fever with thrombocytopenia syndrome virus [4-6]. Since ticks are small, their dispersal is closely related to the movement of their hosts [7-8]. The dynamics of tick-borne pathogens are influenced by the habitat distribution and dispersal behaviors of vectors and hosts along environmental gradients [9]. Therefore, understanding the complex interaction between these factors is important in understanding the spread of tick-borne diseases in Japan.

Tick population genetic analysis provides data that help identify the dispersal pattern of ticks based on gene flow between local populations [10]. Tick dispersal of many three-host Ixodid ticks depends on the movements of their vertebrate hosts, which influences each tick's potential to spread its pathogen [7-8]. For example, contrasting patterns in the population genetic structures of *I. ovatus* and *H. flava* in the Niigata Prefecture of Japan suggest that host mobility during the immature stages of tick development may influence the genetic structure of adult ticks by affecting survivability into their adult stages [11-12]. *I. ovatus* populations had greater genetic divergence possibly due to the limited dispersal of their small mammalian hosts during the immature development stage; *H. flava* populations showed a more homogenized structure possibly due to the larger mobility of their large mammalian hosts and avian-mediated dispersal [11]. Other studies have also revealed low gene flow in ticks with low-mobility hosts (e.g., small mammals) and higher gene flow in ticks with highly mobile hosts (e.g., large mammals and birds) [10; 13-16].

The spatial distribution and movement of the vector (i.e., ticks) may determine the spatial distribution of the pathogen's (i.e., *Rickettsia*) infection rate [17]. Previous studies have shown that the pathogen infection rate can be influenced by many factors, such as the vector's genetic diversity, gene flow, and spatial structure [5-6; 11; 18-25]. For example, previous studies have shown that strong gene flow between local vector populations tends to reduce the spatial heterogeneity of pathogen infection rates between populations [26-27]. Thus, the spatial distribution and movement of the vector may affect the spatial distribution of the pathogen. To our knowledge, no previous studies have examined the relationship between the spatial heterogeneity of *Rickettsia* infection rates and population genetic structure in ticks.

Environmental factors may relate to the population genetic structure of ticks [28-29], with limited gene flow increasing genetic variation between populations along an altitudinal gradient, as reported in several studies on other species [30-32]. Thus, in this study, we expected to see a highly divergent population genetic structure in *I. ovatus* along an altitudinal gradient due to the limited movement of their mammalian hosts along that gradient; whereas *H. flava* should show a less divergent structure along the altitudinal gradient due to the higher mobility of its hosts. To our knowledge, no studies have focused on tick gene flow along an altitudinal gradient.

In the study by [11], no significant influence of environmental factors, including altitude, was observed in the genetic structures of *I. ovatus* and *H. flava* based on the mantel test, but the study did not use any other robust analytical methods to thoroughly examine the influence of altitude on tick genetic structure. In another study, major spotted fever group *Rickettsia* (SFGR) prevalence was analyzed in a total of 3,336 immature and adult ticks across the Niigata Prefecture, Japan in the following tick species: *Dermacentor taiwanensis*, *Haemaphysalis flava*, *Haemaphysalis hystricis*, *Haemaphysalis longicornis*, *Haemaphysalis megaspinosa*, *Ixodes columnae*, *Ixodes monospinosus*, *Ixodes nipponensis*, *Ixodes ovatus*, and *Ixodes persulcatus* [6].

Three SFGR species namely *Rickettsia asiatica, R. helvetica* and *R. monacensis* were detected in *H. flava, Haemaphysalis longicornis, Ixodes monospinus, Ixodes nipponensis*, and *Ixodes ovatus*, no spatial distribution of *Rickettsia* infection rates was found among the local populations. To our knowledge, no previous studies have considered the influence of environmental factors on tick population genetic structure or the spatial distribution of *Rickettsia* infection rates along an altitudinal gradient in local tick populations.

Here, we determine the relationship between the vector tick population genetic structure and *Rickettsia* infection rates as influenced by an altitudinal gradient. Based on the isolation by environment (IBE) theory, genetic differentiation increases with environmental variation, regardless of geographic distance [33-35]. Thus, we hypothesized that limited gene flow among tick populations along an altitudinal gradient might cause spatially heterogenous *Rickettsia* infection rates in ticks. The present study used cytochrome oxidase 1 (*cox1*) mitochondrial gene sequences and *Rickettsia*-infected and uninfected data for *I. ovatus* (n = 307) and *H. flava* (n = 220) individuals collected in 2016–2017 as reported in [6; 11]. Additionally, we added new *cox1* sequences and *Rickettsia*-infected/uninfected ticks from *I. ovatus* (n= 39)and *H. flava* (n=23) individuals and five other species collected in 2018.

2. Materials and Method

Published data of [6;11]

In this study, we used *cox1* sequence data from [11] for *I. ovatus* (n = 307) and *H. flava* (n = 220) ticks collected from April 2016 to November 2017 from 30 sites across the Niigata Prefecture, Japan. *Rickettsia* infection rate data were obtained from [6] from *I. ovatus* (n = 29), *H. flava* (n = 2), *I. monospinus* (n = 4), and *I. nipponensis* (n = 2) from 38 sites across Niigata

Prefecture. The 38 sites surveyed in the previous study by [6] include the 30 sites that were used in this present study (S1 Table). Please refer to [6;11] for more information about the study sites, collection, sampling identification, DNA extraction, PCR amplification, and sequencing methods used in each respective study.

To strengthen our analysis, we also added data collected from April to October 2018 from additional individuals (n=62;) sampled at 30 sites across the Niigata Prefecture, including two sites not previously sampled by [11]. At these sites, ticks were collected 2–14 times from six core sites among the 30 sites, while ticks were collected once at the remaining sites. The altitude at each site ranged from 8 to 1402 meters above sea level (m.a.s.l.), with a mean altitude of 348 m.a.s.l.

Unpublished data from 2018 collection

Ticks collected were stored at 4 °C in microcentrifuge tubes with 70% ethanol. Each collected tick was morphologically identified using a stereo microscope following the identification keys of [36]. Genomic DNA was extracted using Isogenome DNA extraction kits (Nippon Gene Co. Ltd. Tokyo, Japan) following the manufacturer's recommended protocol.

In this study, we combined previously published data from [6] with our newly collected data to calculate the *Rickettsia* infection rate, which is the percentage of *Rickettsia*-infected ticks from each obtained population. We analyzed the obtained tick DNA for spotted fever group *Rickettsia* (SFGR) detection and host identification and amplified the mitochondrial gene *cox1* for population genetic analysis. We performed nested PCR targeting the following genes for the detection of *Rickettsia* sp.: 17-kDA antigen gene (17-kDA); citrate synthase gene (gltA); spotted fever group (SFG)-specific outer membrane protein A gene (rOmpA); and outer membrane protein B gene (rOmpB) as described and analyzed in [6]. Briefly, we first amplified the 17-kDa protein. If

the results were positive, then PCR was performed to target gltA. Samples that were positive with both 17-kDA and gltA were regarded as positive for SFGR and a nested PCR was performed to target the rOmpA and rOmpB gene, samples that are positive for 17-Kda, gltA, rOmpA, and rOmpB genes were sequenced to identify the *Rickettsia* species. The amplified PCR products were purified using AMPure XP (Beckman Coulter Co., Japan) and sequenced using the Big Dye Terminator Cycle Sequence Kit (Thermo Fisher Scientific).

The *cox1* mitochondrial gene was amplified by PCR for *cox1* (658 base pairs) using the primer pairs LCO-1490 (5′- AAACTTCAGGGTGACCAAAAAATCA-3') and HCO1–2198 (5′–AAACTTCAGGGTGACCAAAAAATCA - 3) for phylogenetic analysis and tick species identification [37]. The PCR amplification profile included an initial denaturation of 94 °C for 2 min, followed by denaturation at 94 °C for 30 s, then annealing at 38 °C for 30 s, followed by an extension of 72 °C for 1 min for 30 cycles, and a final extension of 72 °C for 10 min. The obtained PCR products were purified using the QIAquick 96 PCR Purification Kit (Qiagen, Germany) following the manufacturer's instructions and were sequenced by Eurofin Genomics, Inc. (Tokyo, Japan).

Each forward and reverse read was assembled using CodonCode Aligner version 1.2.4 software (https://www.codoncode.com/aligner/). Low-quality bases were removed in the aligned sequences, and no ambiguous bases were detected. We used the MAFFT alignment online program (https://mafft.cbrc.jp/alignment/server/) to perform multiple alignments using the default settings. The sequences were checked for similarities with the deposited reference sequences from GenBank for sequence quality and tick species confirmation using BLAST (https://blast.ncbi.nlm.nih.gov/Blast.cgi). All final aligned sequences were checked in Mesquite version 3.5 [38]. The protein-coding genes were translated to amino acids to confirm the absence of stop codons.

Population genetic analysis

Multiple sites that are in proximity to each other were combined for population genetic analysis if less than eight individuals were obtained per site, which resulted in 8 populations labeled A to H (S1 Table). Three sites were excluded from the population genetic analysis because of the limited number of obtained individuals (<8) and the lack of a nearby site to combine into a single population.

The final *cox1* sequences of the tick species: *I. ovatus* and *H. flava* were individually analyzed using DNASp version 6.12.03 to determine the haplotype diversity per species [39]. The level of genetic divergence between each population was quantified per species using global F_{ST}. Significance was tested using Arlequin software version 3.5.2.2 [40] with 9999 permutations.

The genetic relationship between the *I. ovatus* populations was visualized using the unweighted pair group with the arithmetic mean (UPGMA) cluster method using the APE package [41] for the RStudio software (R Development Core Team, 2016). A cluster dendrogram was created using pairwise F_{ST} values genetic distance matrix from GenAlEx.

Haplotype network and phylogenetic analyses

We constructed a haplotype network analysis using PopART program version 1.7 (http://popart.otago.ac.nz/index.shtml) on *cox1 I. ovatus* and *H. flava* sequences to assess haplotype relationships using the median-joining network algorithm [42]. Briefly, we constructed a Bayesian phylogenetic tree of *cox1* haplotypes for *I. ovatus* and *H. flava,* respectively, using BEAST version 1.10.14 [43]. We used the Hasegawa-Kishino-Yano substitution model with estimated base frequencies. We employed a strict clock model and used the coalescent prior as the tree prior. A maximum clade credibility tree was acquired using TreeAnnotator version 1.10.14 using trees from

BEAUti version1.10.14 with 90% of the trees as the burn-in. We viewed the constructed maximum clade credibility tree using FigTree version 1.4.4.

Statistical analysis

To determine whether there was a significant difference in the *Rickettsia* infection rate between haplotype groups for *I. ovatus* and *H. flava,* we performed a z-score test at $p < 0.05$. To determine whether there were differences in the mean altitude between the haplotype groups, we used the Welch t-test at $p < 0.05$.

3. Results

The total number of positive (pos) and negative (neg) ticks for *Rickettsia* infections from the ticks collected in 2018 were: *I. ovatus* (neg = 22, pos = 17); *H. flava* (neg = 10, pos = 13); *I. monospinus* (neg = 11, pos = 6); *I. asanumai* (neg = 4, pos=5); *I. nipponensis* (pos = 4); *I. persulcatus* (neg = 4); and *H. japonica* (pos = 2). In this study, the total number of samples from the previously published data [6;11] and the unpublished data from 2018 were: *I. ovatus* (n = 346); *H. flava* (n = 243); *I. monospinus* (n = 19); *I. asanumai* (n = 9); *I. nipponensis* (n = 4); *I. persulcatus* (n = 4); and *H. japonica* (n = 2). **T**he number of adult ticks whose *cox1* was successfully sequenced per species were: *I. ovatus* (346); *H. flava* (243); *I. monospinus* (19); *I. asanumai* (9); *I. nipponensis* (4); *I. persulcatus* (4); and *H. japonica* (2) (Table 1).

Table 1. Summary of the haplotype and *Rickettsia* infection rates among the 7 ixodid tick species obtained in the Niigata Prefecture, Japan

Tick species ***	ns	n	nh	r	Global F_{ST}
1. *Ixodes ovatus*	30	346	59	46 (13.26%)	0.4154*
2. *Haemaphysalis flava*	18	243	66	15 (6.17%)	0.3597
3. *Ixodes monospinus*	6	19	9	6 (31.58%)	0.9034
4. *Ixodes asanumai*	7	9	4	5 (55.55%)	1.0000
5. *Ixodes nipponensis*	2	4	4	4 (100%)	0.3345
6. *Ixodes persulcatus*	3	4	3	0 (0%)	1.0000
7. *Haemaphysalis japonica*	2	2	2	2 (100%)	**
**Total**		628		78	

Abbreviations: ns no. of sampling sites; n sample size; nh no of haplotypes; r *Rickettsia* infection rate per species; *p < 0.05** not enough samples for analysis***tick species identification is based on molecular identification using the *cox1* marker and BLAST results

We detected SFGR in 78 (12.44%) out of 627 ixodid ticks, with the highest detected in *I. ovatus* (46/346; 13.29%), *H. flava* (15/243; 6.17%), and *I. monospinus* (6/19; 31.58%) as summarized in Table 1. Out of the 46 *Rickettsia*-infected *I. ovatus* ticks, 25 displayed a 100% identity match with *Rickettsia asiatica* in the 17kDA, *gltA,* and *rOmpB* markers [6], while an additional 19 adult *I. ovatus* from the 2018 collection were also positive with *R. asiatica.* Two haplotypes were found in the rOmpB and 17kDA markers, respectively. One haplotype was found in only one individual (17369). Two out of the 15 *Rickettsia*-infected *H. flava* ticks were found to have the same haplotypes in the 17Kda, gltA,and rOmpA markers, and were identified as *Rickettsia sp.* (LC461063). The remaining 13 *Rickettsia*-infected *H. flava* ticks were identified as *Rickettsia sp.* Four out of the six *Rickettsia*-infected *I. monospinus* were identified as *Rickettsia helvetica* with the same haplotype found in the 17kDA, gltA, and rOmpB markers.

Based on the population genetic analysis of the *cox1* sequences, we found a significant global F_{ST} of 0.4154 at $p < 0.05$ for *I. ovatus* (Table 1). In contrast, no significant global F_{ST} values

were observed in the *H. flava, I. monospinus, I. asanumai, I. nipponensis,* or *I. persulcatus* samples (Table 1). There were 59 and 66 *cox1* haplotypes found among the 346 *I. ovatus* and 243 *H. flava* individuals, respectively. We found the following number of *cox1* haplotypes in the remaining species: *I. monospinus* (n = 9), *I. asanumai* (n = 4), *I. nipponensis* (n = 4), *I. persulcatus* (n = 3), and *H. japonica* (n = 2).

The *cox1* haplotype network of *I. ovatus* (Figure 1) revealed four genetic groups, wherein three genetic groups (1, 2, and 3) were distributed along different altitudinal gradients, as shown in Figure 2. These four genetic groups were concordant with the four clusters found in the *I. ovatus* phylogenetic tree (S1 Figure). The habitat distribution of genetic group 3 was limited to high altitude sites only (range = 16–912 m.a.s.l.), whereas genetic groups 1 (255–471 m.a.s.l.) and 2 (84–1354 m.a.s.l.) were distributed at lower altitudes (Figure 2). The Welch t-test revealed a significant difference between the mean altitudes of genetic groups 1 and 3 at $p < 0.05$ (Table 2); however, no significant difference was observed between genetic groups 1 and 2 or groups 2 and 3. We found a significant difference in the *Rickettsia* infection rates between *I. ovatus* genetic groups 1 and 2 based on the z-score test, but no significant difference between groups 1 and 3 or groups 2 and 3 (Table 2). The mean altitude between the *Rickettsia*-infected (=273.72 m.a.s.l.) and non-infected *I. ovatus* (=369.61 m.a.s.l.) revealed a significant difference based on the Welch t-test at $p < 0.05$ (Figure 3). The UPGMA dendrogram of *I. ovatus* revealed two genetic clusters, 1 and 2 using the genetic distance among the seven populations excluding one population due to the limited number of samples (Figure 4).

Figure 1. Median-joining network of the 59 *cox1* haplotype sequences of *Rickettsia* positive and negative *I. ovatus.* Haplotype groups are indicated by numbers (1 to 4).

Figure 2. The influence of altitude on the habitat distribution of *I. ovatus*. The points indicate the sampling sites of the collected individuals per *I. ovatus* haplotype group (n = 3) in the haplotype network (Figure 1). The map shows the elevation level across the sampling area, with Niigata Prefecture Japan depicted as a color gradation. Group 4, not shown in this figure, with haplotypes 54 and 55 was found on Sado island, which is encircled in black in box 3.

Figure 3. The relationship between the mean altitude of *Rickettsia* positive (n = 46) and negative (n = 300) *I. ovatus.* Welch t-tests revealed a significant difference in the mean altitude between *Rickettsia-infected* and non-infected *I. ovatus* at $p < 0.05$. Populations with combined sites are labeled A to H.

Figure 4. An unweighted pair group method with the arithmetic mean (UPGMA) dendrogram of *I. ovatus* based on the pairwise genetic distance (F_{ST}) of *cox1* among the 7 populations across Niigata Prefecture, Japan. We excluded one population due to the limited number of samples.

Table 2. The differences in *Rickettsia* infection rates and mean altitude in *I. ovatus* haplotype groups and cluster dendrogram groups. The table shows the distribution of *Rickettsia*-infected and uninfected *I. ovatus* and the mean altitude in each of the haplotype groups as shown in Figure 1 *(I. ovatus* haplotype network) and the cluster dendrogram in Figure 4 (*I. ovatus* cluster dendrogram). The z-score test showed a significant difference at $p < 0.05$ between the *Rickettsia* detection rates in haplotype groups 1 and 2 (indicated by [ab)] and in cluster dendrogram groups 1 and 2. The Welch t-test at $p < 0.05$ revealed a significant difference in the mean altitude of haplotype groups 1 and 3

and cluster dendrogram groups 1 and 2 (indicated by [ab]). Haplotype group 4 was not included in the analysis due to its low sample size.

**Haplotype group**	***Rickettsia* infection rate**			**Mean altitude**
	Positive	Negative	Detection rate	
**1**	1	92	1.07%[ab]	294[ab]
**2**	28	99	22.04%[ab]	341[a]
**3**	16	110	12.70%[a]	374[ab]
**Dendrogram Group**				
**1**	4	120	3.23%[ab]	30
**2**	40	227	14.98%[ab]	345

The *cox1* haplotype network of *H. flava* displayed two genetic groups (S2 Figure) consistent with the *H. flava* phylogenetic tree (S3 Figure). Based on the Welch t-test, we found no significant differences in mean altitude (205 and 165 m.a.s.l.) at $p < 0.05$ between the two *H. flava* genetic groups (S3 Table). No significant difference between the *Rickettsia* infection rates of *H. flava* genetic groups 1 and 2 was observed using the z-score test at $p < 0.05$ (S2 Table).

4. Discussion

Our findings support our hypothesis that a genetically structured tick population, such as *I. ovatus*, can cause the *Rickettsia* infection rate to be spatially heterogenous due to limited gene flow

along an altitudinal gradient. The significant global F_{ST} estimate of 0.4154 among the *I. ovatus* populations revealed genetic differentiation between the populations as supported by the occurrence of two genetic clusters in the cluster dendrogram. Our results were consistent with our previous study [11] which suggested that the low mobility of the host species for immature *I. ovatus* contributed to low gene flow in the tick populations. The low *I. ovatus* gene flow along the altitudinal gradient might have caused the spatial heterogeneity of *Rickettsia* infection rates among these populations, which is supported by the significant difference found in *Rickettsia* infection rates between genetic clusters 1 and 2. A similar pattern was observed in the studies of [44-46] which found that the infection rate of *Borrelia burgdorferi*, the causative agent of Lyme disease, decreased in ticks along the altitudinal gradient. Low gene flow can cause infected and uninfected ticks to have limited opportunities to traverse a wider spatial area thus causing a heterogeneous *Rickettsia* infection rate [47-48].

We found two genetic groups in the *H. flava* haplotype network, but no significant difference in the *Rickettsia* infection rates between the two groups. These results might be due to the high gene flow observed in the *H. flava* populations, which enable *Rickettsia*-infected and uninfected *H. flava* individuals to traverse between the study sites. The high mobility of the large mammalian hosts used by adult *H. flava* and avian-mediated dispersal during their immature stage probably contributed to their homogenized population genetic structure [11], and the resulting homogenized *Rickettsia* infection rates. Large mammalian hosts and birds have a wide dispersal range that enables the broader movement of *Rickettsia*-infected ticks, as observed in previous studies of *Amblyomma Americanum* [49-51], *H. flava* [11], and *I. ricinus* [52]. Birds are especially good at dispersing over large areas since they can easily traverse landscape barriers such as mountains, fences, glaciers, and oceans that would be difficult for mammals to cross [53].

In addition to host mobility, environmental factors can influence the population genetic structure of ticks [54]. Each tick species has preferred environmental conditions that are conducive to completing the tick life cycle, thus influencing the geographical distribution of ticks and the risk areas for tick-borne diseases [55-56]. Altitude may influence the population genetic structure of ticks through the effect of altitude on the distribution and abundance of ticks and/or their hosts [57-59]. Altitudinal differences between populations can affect genetic divergence [60], through, for example, ecological isolation, which causes natural selection against maladapted immigrants and limits gene flow [61-62]. For example, organisms adapted to low altitudinal sites that cannot tolerate the lower temperatures at higher altitudes would not survive if they dispersed to those higher altitude sites, thus restricting gene flow across the altitudinal gradient [60].

The difference in mean altitude between *I. ovatus* cluster groups 1 and 2 might be due to adaptive divergence along the altitudinal gradient. Individuals from cluster group 1 were distributed in higher altitude areas of the northern mountainous area of Niigata Prefecture, while cluster group 2 individuals were found in the lower altitude regions of northern Niigata Prefecture. Populations along an altitudinal gradient are prone to differentiating selection pressures, which result in local adaptation [63]. Altitudinal gradients may also cause the varying ambient temperature, precipitation, and humidity levels essential to ticks' development and survival [64]. These varying environmental factors may cause ticks to have difficulty dispersing over a wide habitat range. Thus, ticks may need to adapt to extreme habitats, such as extreme altitude or precipitation levels, for survival. For example, *Rhipicephalus compositus* were found in altitudes of 1000–2500 m, but optimal conditions were between 1200–2500 m [64-66].

The different *Rickettsia* infection rates and altitudinal ranges between the *I. ovatus* phylogenetic groups may be explained by adaptive evolutionary theory, which states that organisms adjust to new or severe changes in their environment to become better suited to their habitat[67-

68]. Based on the relationship between the *I. ovatus* phylogenetic groups and their mean attitudes*,* *I. ovatus* might be undergoing local adaptation along the altitudinal gradient due to the higher genetic differentiation between populations as supported by the significant global F_{ST} (0.4154) found in *I. ovatus*. Based on isolation by environment (IBE), genetic differentiation will increase with increased environmental differences independent of geographic distances [33-34;69]. When environmental conditions differ, the success of immigration in a new habitat is reduced, which may increase the genetic fixation rate due to a lower chance of outcrossing; thereby enhancing genetic isolation [70]. Thus, the lower gene flow along the altitudinal gradient reduced the spatial homogeneity of *Rickettsia* infection rates among the *I. ovatus* tick populations, thus causing the different *Rickettsia* infection rates obtained.

In this study, only a few (n < 20) *I. monospinus, I. asanumai, I. nipponensis* and *H. japonica* were collected; however, a high *Rickettsia* infection rate (31.58%–100%) was found despite these limited numbers. In previous studies, high *Rickettsia* infection rates despite a few individuals were also observed in *I. monospinus* in several prefectures in Japan [71-73]. The extent of tick habitat distribution may vary between species, which may have influenced the widely different *Rickettsia* infection rates observed among the species in this study. The high *Rickettsia* infection rate in these ticks is probably due to effective transovarial transmission [74]. Furthermore, *Rickettsial* endosymbionts are inclined to have a high infection rate in some tick species populations because they contribute to the tick microbiome which might imply nutritional symbiosis in the tick species with high *Rickettsia* infection despite the low number of samples tested [75-77]. Additionally, the few collected individuals of these species may be habitat specialists that thrive in a narrow habitat range in contrast to *I. ovatus* and *H. flava*, which may be habitat generalists capable of thriving in a wide habitat range [78]. The narrow habitat range of habitat specialist ticks might have enabled increased interactions between infected and uninfected *Rickettsia* ticks within the small spatial scale

compared to the habitat generalist ticks, thus causing their high *Rickettsia* infection rates. This conclusion should be considered with caution, since the limited number of individual ticks collected across the Niigata Prefecture may have caused a bias in the estimated *Rickettsia* infection rates determined in this study. We suggest conducting future research on *Rickettsia* infection rates in a wider sampling range with an increased number of *I. monospinus, I. asanumai, I. nipponensis*, and *H. japonica* individuals to further increase our understanding of the association between *Rickettsiae* and their tick vectors.

The occurrence of local adaptation in tick populations could affect the future of the tick-borne disease landscape. Environmental factors, such as precipitation, temperature, and altitude, have been shown to drive population differentiation in insects, such as *Anopheles* mosquitoes and *Drosophila* flies [79-81], but studies on the environmental adaptation of ixodid ticks, such as *I. ovatus*, and its *Rickettsia* infection susceptibility, have not yet been performed. [7] suggested that environmental conditions that affect bird hosts can also affect the local adaptation of ticks. Few studies have assessed such local adaptation in multiple organisms with varying dispersal abilities [82-86] and are an area in need of future research.

One of the limitations of this study is the use of one mitochondrial gene *cox1*, despite this, we were able to determine the relationship between the tick population genetic structure and *Rickettsia* infection rates as influenced by the altitudinal gradient. The mitochondrial *cox1* gene has been widely used for population genetic analysis of many tick species and was proven to be informative in determining the relationship from the subfamily to the population level [87-91]. Mitochondrial genes have a mutation rate that is useful in species-level phylogenetics and can be used for wide geographic ranges however its resolution is not fine enough to study species selection [10]. In future studies, we suggest including additional mitochondrial genes and or nuclear genes.

Since ticks are blood-sucking ectoparasites, they directly influence their mammalian hosts and the pathogens they transmit [94-96]. The interaction between the vector (tick), host, and pathogen (*Rickettsia*) is essential in understanding and predicting the risk and transmission of tick-borne diseases [97]. Understanding the genetic structure of ticks can serve as an alternative indicator to infer the potential spread of its pathogen [98]. Our study found relationships between (1) the population genetic structure of ticks and the corresponding *Rickettsia* infection rates, (2) altitude and the population genetic structure of ticks, and (3) altitude and *Rickettsia* infection rates. We found that host mobility may influence the genetic structure of ixodid ticks. This information can be used to design more effective tick-borne disease control programs that focus on screening and detecting pathogens found in ticks and their mammalian hosts. For example, patterns of disease transmission from ticks with a high genetic divergence and less mobile hosts, such as *I. ovatus*, are likely due to the movement of infected hosts rather than infected ticks. Thus, screening prospective tick hosts for *Rickettsia* infection would be more suitable in this example. We suggest screening the hosts of immature *I. ovatus*, such as small rodents, instead of screening ticks. The *Rickettsia* infection rate in tick genetic groups can predict the spread of tick-borne diseases caused by *Rickettsia,* such as the Japanese spotted fever. We also found that altitude may influence the *Rickettsia* infection rate of *I. ovatus* genetic groups. This information can be used to determine the high-risk areas (e.g., lowland, mountains, etc.) of tick-borne diseases along an altitudinal gradient. Genetically structured arthropod vectors, such as ticks, can have different vector competencies, and environmental factors, such as altitudinal gradients, that can influence the vector's ability to acquire, transmit, and maintain the pathogen infection [99].

Acknowledgments

The authors are thankful for the assistance from the Niigata Prefectural Office during the tick sampling collection. We would also like to thank the alumni of MECOH Lab Ehime University for

their help in molecular analyses: Masaya Doi, Kohki Tanaka, and Mizuki Ueda. We are also grateful to Micanaldo Francisco for the construction of the map for this manuscript. We would like to thank Enago (www.enago.jp) for the English language review and proofreading of the manuscript.

References

1. Neumann M. Eine hysterische Hausepidemie. European Neurology. 1899;5(6):405-10.
2. Miyamoto, K., Nakao, M., Fujita, H., Sato, F., 1993. The ixodid ticks on migratory birds in Japan and the isolation of Lyme disease spirochetes from bird-feeding ticks. Med. Entomol. and zool., 44(4), 315-326. https://doi.org/10.7601/mez.44.315
3. Neumann H. Zur Behandlung der Pleuritis exsudativa. DMW-Deutsche Medizinische Wochenschrift. 1897 Jan;23(05):73-5.
4. Yu XJ, Liang MF, Zhang SY, Liu Y, Li JD, Sun YL, Zhang L, Zhang QF, Popov VL, Li C, Qu J. Fever with thrombocytopenia associated with a novel bunyavirus in China. N. Engl. J. Med. 2011 Apr 21;364(16):1523-32. https://doi.org/10.1056/nejmoa1010095
5. Yamaji K, Aonuma H, Kanuka H. Distribution of tick-borne diseases in Japan: Past patterns and implications for the future. J. Infect. Chemother.2018 Jul 1;24(7):499-504. https://doi.org/10.1016/j.jiac.2018.03.012
6. Arai R, Sato M, Kato M, Aoki J, Nishida A, Watanabe K, Hirokawa C, Ikeda S, Watanabe K, Regilme MA, Sato MO. Spotted fever group rickettsiae (SFGR) detection in ticks following reported human case of Japanese spotted fever in Niigata Prefecture, Japan. Sci. Rep. 2021 Jan 28;11(1):2595. https://doi.org/10.1038/s41598-021-81587-9

7. McCoy KD, Boulinier T, Tirard C, Michalakis Y. Host specificity of a generalist parasite: genetic evidence of sympatric host races in the seabird tick *Ixodes uriae*. J. Evol. Biol.. 2001 May 9;14(3):395-405. https://doi.org/10.1046/j.1420-9101.2001.00290.x

8. Oliver JD, Bennett SW, Beati L, Bartholomay LC. Range expansion and increasing *Borrelia burgdorferi* infection of the tick *Ixodes scapularis* (Acari: Ixodidae) in Iowa, 1990–2013. J. Med. Entomol. 2017 Nov 7;54(6):1727-34. https://doi.org/10.1093/jme/tjx121

9. Agany, D. D., Pietri, J. E., Gnimpieba, E. Z., 2020. Assessment of vector-host-pathogen relationships using data mining and machine learning. Comput. Struct. Biotechnol. J., 18, 1704-1721. https://doi.org/10.1016/j.csbj.2020.06.031

10. Araya-Anchetta A, Busch JD, Scoles GA, Wagner DM. Thirty years of tick population genetics: a comprehensive review. Infect. Genet. Evol. 2015 Jan 1;29:164-79. https://doi.org/10.1016/j.meegid.2014.11.008

11. Regilme MA, Sato M, Tamura T, Arai R, Sato MO, Ikeda S, Gamboa M, Monaghan MT, Watanabe K. Comparative population genetic structure of two ixodid tick species (Acari: Ixodidae)(*Ixodes ovatus* and *Haemaphysalis flava*) in Niigata prefecture, Japan. Infect. Genet. Evol. 2021 Oct 1;94:104999.

12. Estrada-Peña AJ. Ticks as vectors: taxonomy, biology and ecology. Rev. Sci. Tech. 2015 Apr 1;34(1):53-65. https://doi.org/10.20506/rst.34.1.2345.

13. Mixson TR, Lydy SL, Dasch GA, Real LA. Inferring the population structure and demographic history of the tick, *Amblyomma americanum* Linnaeus. J. Vector Ecol. 2006 Jun;31(1):181-92. https://doi.org/10.3376/1081-1710(2006)31[181:itpsad]2.0.co; 2.

14. Trout RT, Steelman CD, Szalanski AL. Population genetics of *Amblyomma americanum* (Acari: Ixodidae) collected from Arkansas. J. Med. Entomol. 2014 Dec 1;47(2):152-61. https://doi.org/10.1603/me09106

15. Guglielmone AA, Nava S, Mastropaolo M, Mangold AJ. Distribution and genetic variation of *Amblyomma triste* (Acari: Ixodidae) in Argentina. Ticks Tick Borne Dis. 2013 Sep 1;4(5):386-90. https://doi.org/10.1016/j.ttbdis.2013.01.009

16. Lampo M, Rangel Y, Matat A. Population genetic structure of a three-host population genetic in *Amblyommma dissimile*. J. Parasitol. 2015;84:1137-42.

17. Dougherty ER, Seidel DP, Carlson CJ, Spiegel O, Getz WM. Going through the motions: incorporating movement analyses into disease research. Ecol. Lett. 2018 Apr;21(4):588-604. https://doi.org/10.1111/ele.12917

18. Regoes RR, Nowak MA, Bonhoeffer S. Evolution of virulence in a heterogeneous host population. Evol. 2000 Feb;54(1):64-71. https://doi.org/10.1111/j.0014-3820.2000.tb00008.x

19. Boots M, Mealor M. Local interactions select for lower pathogen infectivity. Sci. 2007 Mar 2;315(5816):1284-6.https://doi.org/10.1126/science.1137126

20. Lion S, Gandon S. Evolution of spatially structured host–parasite interactions. Journal of Evol. Biol. 2015 Jan;28(1):10-28. https://doi.org/10.1111/jeb.12551

21. Chin KM, Wolfe MS. Selection on *Erysiphe graminis* in pure and mixed stands of barley. Plant Pathol. 1984;33:535-546. doi: 10.1111/j.1365-3059.1984.tb02878.x

22. Thrall PH, Burdon JJ. Evolution of virulence in a plant host-pathogen metapopulation. Science. 2003;299:1735-1737. doi: 10.1126/science.1080070.

23. White PS, Choi A, Pandey R, Menezes A, Penley M, Gibson AK, de Roode J, Morran L. Host heterogeneity mitigates virulence evolution. Biol Lett. 2020;16:20190744. doi: 10.1098/rsbl.2019.0744.

24. Gibson AK, Baffoe‐Bonnie H, Penley MJ, Lin J, Owens R, Khalid A, Morran LT. The evolution of parasite host range in heterogeneous host populations. J Evol Biol. 2020;33:773-782. doi: 10.1111/jeb.13608.

25. Bono LM, Gensel CL, Pfennig DW, Burch CL. Competition and the origins of novelty: experimental evolution of niche-width expansion in a virus. Biol Lett. 2013;9:20120616. doi: 10.1098/rsbl.2012.0616.

26. Streicker DG, Winternitz JC, Satterfield DA, Condori-Condori RE, Broos A, Tello C, Recuenco S, Velasco-Villa A, Altizer S, Valderrama W. Host–pathogen evolutionary signatures reveal dynamics and future invasions of vampire bat rabies. Proc Natl Acad Sci U S A. 2016;113:10926-10931. doi: 10.1073/pnas.1606587113.

27. Fountain‐Jones NM, Craft ME, Funk WC, Kozakiewicz C, Trumbo DR, Boydston EE, Lyren LM, Crooks K, Lee JS, VandeWoude S, Carver S. Urban landscapes can change virus gene flow and evolution in a fragmentation‐sensitive carnivore. Mol Ecol. 2017;26:6487-6498. doi: 10.1111/mec.14375.

28. Daniel M, Malý M, Danielová V, Kříž B, Nuttall P. Abiotic predictors and annual seasonal dynamics of *Ixodes ricinus*, the major disease vector of Central Europe. Parasit Vectors. 2015;8:1-12. doi: 10.1186/s13071-015-1092-y.

29. Eisen RJ, Eisen L, Beard CB. County-scale distribution of *Ixodes scapularis* and *Ixodes pacificus* (Acari: Ixodidae) in the continental United States. J Med Entomol. 2016;53:349-386. doi: 10.1093/jme/tjv237.

30. Chapman MA, Hiscock SJ, Filatov DA. Genomic divergence during speciation driven by adaptation to altitude. Mol Biol Evol. 2013;30:2553–2567. doi: 10.1093/molbev/mst168.

31. Osborne OG, Batstone TE, Hiscock SJ, Filatov DA. Rapid speciation with gene flow following the formation of Mt. Etna. Genome Biol Evol. 2013;5:1704–1715. doi: 10.1093/gbe/evt127.

32. Polato NR, Gray MM, Gill BA, Becker CG, Casner KL, Flecker AS, Kondratieff BC, Encalada AC, Poff NL, Funk WC, Zamudio KR. Genetic diversity and gene flow decline with elevation in montane mayflies. Heredity. 2017 Aug;119(2):107-116. doi: 10.1038/hdy.2017.19. PMID: 28333156.

33. Wang ,J., Summers,K., 2010. Genetic structure is correlated with phenotypic divergence rather than geographic isolation in the highly polymorphic strawberry poison-dart frog. Mol. Ecol., 19, 447–458. https://doi.org/10.1111/j.1365-294x.2009.04465.x

34. Bradburd, G.S., Ralph, P.L., Coop, G.M., 2013. Disentangling the effects of geographic and ecological isolation on genetic differentiation. Evolution, 67, 3258–3273. https://doi.org/10.1111/evo.12193

35. Sexton,J.P., Hangartner,S.B., Hoffmann,A.A., 2014. Genetic isolation by environment or distance: which pattern of gene flow is most common?. Evol., 68, 1–15. https://doi.org/10.1111/evo.12258

36. Yamaguti, N., Tipton, V. J., Keegan, H. L., & Toshioka, S. (1971). Ticks of Japan, Korea, and the Ryukyu islands. Brigham Young University Science Bulletin, Biological Series, 15, 1.

37. Vrijenhoek R. DNA primers for amplification of mitochondrial cytochrome c oxidase subunit I from diverse metazoan invertebrates. Mol Mar Biol Biotechnol. 1994 Oct 1;3(5):294-9. PMID: 7881515.

38. Maddison, W.P., Maddison, D.R., 2011. Mesquite: A modular system for evolutionary analysis. Available at: http://mesquiteproject.org.

39. Rozas J, Ferrer-Mata A, Sánchez-DelBarrio JC, Guirao-Rico S, Librado P, Ramos-Onsins SE, Sánchez-Gracia A. DnaSP 6: DNA sequence polymorphism analysis of large datasets. Mol Biol Evol. 2017 Dec 1;34(12):3299-3302. doi: 10.1093/molbev/msx248. PMID: 29036515.

40. Excoffier, L., & Lischer, H. E. L. (2010). Arlequin suite ver 3.5: A new series of programs to perform population genetics analyses under Linux and Windows. Molecular Ecology Resources, 10(3), 564-567. https://doi.org/10.1111/j.1755-0998.2010.02847.x

41. Paradis, E., Schliep, K., 2018. APE 5.0: an environment for modern phylogenetics and evolutionary analyses in R. Bioinformatics, 35, 526–528. https://doi.org/10.1093/bioinformatics/bty633.

42. Bandelt, H.J., Forster, P., Röhl, A., 1999. Median-joining networks for inferring intraspecific phylogenies. Mol. Biol. Evol. 16, 37–48. https://doi.org/10.1093/oxfordjournals.molbev.a026036

43. Drummond AJ, Rambaut A. BEAST: Bayesian evolutionary analysis by sampling trees. BMC Evol Biol. 2007 Nov 8;7:214. doi: 10.1186/1471-2148-7-214. PMID: 17996036; PMCID: PMC2222611.

44. Burri, C., Cadenas, F.M., Douet, V., Moret, J., Gern, L., 2007. Ixodes ricinus density and infection prevalence of *Borrelia burgdorferi* sensu lato along a north-facing altitudinal gradient in the Rhône Valley (Switzerland). Vector-Borne and Zoonotic Diseases, 7, 50-58. https://doi.org/10.1089/vbz.2006.0569.

45. Jouda, F., Perret, J.L., Gern, L., 2004. *Ixodes ricinus* density, and distribution and prevalence of *Borrelia burgdorferi* sensu lato infection along an altitudinal gradient. J. Med. Entomol. 41, 162-169. doi: 10.1603/0022-2585-41.2.162.

46. Moran-Cadenas F, Schneider H, Lommano E, Burri C, Moret J, Gern L. A comparison of two DNA extraction approaches in the detection of *Borrelia burgdorferi* sensu lato from live Ixodes ricinus ticks by PCR and reverse line blotting. Vector Borne Zoonotic Dis. 2007 Dec 1;7(4):555-62. https://doi.org/10.1089/vbz.2006.0596

47. Gooding, R.H. Genetic variation in arthropod vectors of disease-causing organisms: Obstacles and opportunities. Clin. Microbiol. Rev. 1996; 9: 301.https://doi.org/10.1128/cmr.9.3.301

48. Páez-Triana, L., Muñoz, M., Herrera, G., Moreno-Pérez, D.A., Tafur-Gómez, G.A., Montenegro, D., Patarroyo, M.A., Paniz-Mondolfi, A., Ramírez, J.D., 2021. Genetic diversity and population structure of *Rhipicephalus sanguineus* sensu lato across different regions of Colombia. Parasit Vectors, 14, 1-11. https://doi.org/10.1186/s13071-021-04898-w

49. Reichard, M.V., Kocan, A.A., Van Den Bussche, A., Barker, R.W., Wyckoff, J., Ewing, S.A., 2005. Sequence variation of the ribosomal DNA second internal transcribed spacer region in two spatially distinct populations of *Amblyomma americanum* (L.)(Acari: Ixodidae). J. Parasitol. 91, 260–264. https://doi.org/10.1645/GE-292R1.

50. Trout, R.T., Steelman, C.D., Szalanski, A.L., 2010. Population genetics of *Amblyomma americanum* (Acari: Ixodidae) collected from Arkansas. J. Med. Entomol. 47, 152–161. https://doi.org/10.1603/me09106.

51. Mixson TR, Lydy SL, Dasch GA, Real LA. Inferring the population structure and demographic history of the tick, *Amblyomma americanum* Linnaeus. J Vector Ecol. 2006 Jun;31(1):181-92. doi: 10.3376/1081-1710(2006)31[181:itpsad]2.0.co; 2.

52. Casati, S., Bernasconi, M.V., Gern, L., Piffaretti, J.C., 2008. Assessment of intraspecific mtDNA variability of European *Ixodes ricinus* sensu stricto (Acari: Ixodidae). Infect. Genet. Evol. 8, 152–158. https://doi.org/10.1016/j.meegid.2007.11.007

53. Hasle, G., 2013. Transport of ixodid ticks and tick-borne pathogens by migratory birds. Front. Cell. Infect. Microbiol., 3, 48. https://doi.org/10.3389/fcimb.2013.00048

54. Eremeeva, M.E. and Dasch, G.A., 2015. Challenges posed by tick-borne rickettsiae: eco-epidemiology and public health implications. Front. Public Health. 3, 55. doi: 10.3389/fpubh.2015.00055.

55. Gage, K.L., Burkot, T.R., Eisen, R.J., Hayes, E.B., 2008. Climate and vector-borne diseases. Am. J. Prev. Med. 35, 436-50. doi: 10.1016/j.amepre.2008.08.030.

56. Parola, P., Raoult, D., 2001. Tick-borne bacterial diseases emerging in Europe. Clin. Microbiol. Infect. 7,80-83. https://doi.org/10.1046/j.1469-0691.2001.00200.x

57. Van Treuren W, Ponnusamy L, Brinkerhoff RJ, Gonzalez A, Parobek CM, Juliano JJ, et al. Variation in the microbiota of Ixodes ticks with regard to geography, species, and sex. Appl Environ Microbiol. 2015;81(18):6200-9. https://doi.org/10.1128/AEM.01562-15.

58. Randolph SE, Miklisová D, Lysy J, Rogers DJ, Labuda M. Incidence from coincidence: patterns of tick infestations on rodents facilitate transmission of tick-borne encephalitis virus. Parasitology. 1999;118:177–186. https://doi.org/10.1017/s0031182098003643.

59. MacDonald AJ, Hyon DW, Iii JBB, Connor KEO, Swei A, Briggs CJ. Lyme disease risk in southern California: abiotic and environmental drivers of *Ixodes pacificus* (Acari: Ixodidae) density and infection prevalence with *Borrelia burgdorferi*. Parasit Vectors. 2017 Jan;10(1):7. https://doi.org/10.1186/s13071-016-1938-y.

60. Funk WC, Murphy MA, Hoke KL, Muths E, Amburgey SM, Lemmon EM, Lemmon AR. Elevational speciation in action? Restricted gene flow associated with adaptive divergence across an altitudinal gradient. J Evol Biol. 2016 Feb;29(2):241-252. https://doi.org/10.1111/jeb.12760.

61. Coyne JA, Orr HA. Speciation. Sunderland, MA: Sinauer Associates; 2004.

62. Nosil P. Ecological Speciation. Oxford University Press; 2012.

63. Lim MC, Bi K, Witt CC, Graham CH, Dávalos LM. Pervasive genomic signatures of local adaptation to altitude across highland specialist Andean hummingbird populations. J Hered. 2021 Mar 1;112(2):229-240. https://doi.org/10.1093/jhered/esab008.

64. Juvenal N, Edward M. Seasonal dynamics and distribution of ticks in Rwanda: implications for tick control strategy in Rwanda. J Anim Vet Adv. 2010;9(1):21-25.

65. Nzige T. Ecologie des tiques du bétail. Rapport de stage, Higher Institute of Agriculture and Animal Husbandry (ISAE-Busogo) Publications, Musanze, Rwanda. 1978.

66. Morel P. Maladies à tiques du bétail en Afrique In: Précis de parasitologie vétérinaire tropicale. Ministére de la Coopération et du Développement, République Française, Norval, R.A.I., Donachie, pp: 471-717. 1981.

67. Kawecki TJ, Ebert D. Conceptual issues in local adaptation. Ecol Lett. 2004 Dec;7(12):1225-1241. https://doi.org/10.1111/j.1461-0248.2004.00684.x.

68. Gregory TR. Understanding natural selection: essential concepts and common misconceptions. Evol Educ Outreach. 2009 Jun;2(2):156-175. https://doi.org/10.1007/s12052-009-0128-1.

69. Wang IJ, Bradburd GS. Isolation by environment. Mol. Ecol. 2014 Dec;23(23):5649-5662. https://doi.org/10.1111/mec.12938.

70. Jiang S, Luo MX, Gao RH, Zhang W, Yang YZ, Li YJ, Liao PC. Isolation-by-environment as a driver of genetic differentiation among populations of the only broad-leaved evergreen shrub *Ammopiptanthus mongolicus* in Asian temperate deserts. Sci Rep. 2019 Jul 30;9(1):1-14. https://doi.org/10.1038/s41598-019-48472-y.

71. Yamauchi T, Takeno K. Record of *Haemaphysalis flava* Neumann (Acari,Ixodidae) from *Zoothera dauma* (Latham) (Aves) in Kyushu, Japan. Esakia. 2000;40:99-101. https://doi.org/10.5109/2647

72. Ishiguro F, Takada N, Fujita H, Noji Y, Yano Y, Iwasaki H. Survey of the vectorial competence of ticks in an endemic area of spotted fever group rickettsioses in Fukui Prefecture, Japan. Microbiol Immunol. 2008;52:305-9. https://doi.org/10.1111/j.1348-0421.2008.00042.x

73. Sato M, Ikeda S, Arai R, Kato M, Aoki J, Nishida A, et al. Diversity and distribution of ticks in Niigata prefecture, Japan (2016–2018): Changes since 1950. Ticks Tick Borne Dis. 2021;12:101683. https://doi.org/10.1016/j.ttbdis.2021.101683.

74. Parola, P., Paddock, C.D., Socolovschi, C., Labruna, M.B., Mediannikov, O., Kernif, T., Abdad, M.Y., Stenos, J., Bitam, I., Fournier, P.E., Raoult, D., 2013. Update on tick-borne rickettsioses around the world: a geographic approach. Clin. Microbiol. Rev., 26, 657-702. doi: 10.1128/cmr.00032-13.

75. Gillespie JJ, Joardar V, Williams KP, Driscoll T, Hostetler JB, Nordberg E, Shukla M, Walenz B, Hill CA, Nene VM, Azad AF. A *Rickettsia* genome overrun by mobile genetic elements provides insight into the acquisition of genes characteristic of an obligate intracellular lifestyle. J Bacteriol. 2012 Jan 15;194(2):376-94. https://doi.org/10.1128/JB.06244-11

76. Clay K, Klyachko O, Grindle N, Civitello D, Oleske D, Fuqua C. Microbial communities and interactions in the lone star tick, *Amblyomma americanum*. Mol Ecol. 2008 Oct;17(19):4371-4381.

77. Budachetri K, Browning RE, Adamson SW, Dowd SE, Chao CC, Ching WM, Karim S. An insight into the microbiome of the *Amblyomma maculatum* (Acari: Ixodidae). J Med Entomol. 2014 Jan;51(1):119-29. doi: 10.1603/me13139. PMID: 24605422.

78. Richmond, C.E., Breitburg, D.L., Rose, K.A., 2005. The role of environmental generalist species in ecosystem function. Ecol. Model. 188, 279-295. doi: 10.1016/j.ecolmodel.2005.03.002.

79. Cheng, C., White, B.J., Kamdem, C., Mockaitis, K., Costantini, C., Hahn, M.W., Besansky, N.J., 2012. Ecological genomics of *Anopheles gambiae* along a latitudinal cline: a population-resequencing approach. Genetics, 190(4), 1417-1432. https://doi.org/10.1534/genetics.112.139899

80. Kapun, M., Barrón, M.G., Staubach, F., Obbard, D.J., Wiberg, R.A.W., Vieira, J., Goubert, C., Rota-Stabelli, O., Kankare, M., Bogaerts-Márquez, M., Haudry, A. (2020). Genomic analysis of European *Drosophila melanogaster* populations reveals longitudinal structure, continent-wide selection, and previously unknown DNA viruses. Mol. Biol. Evol., 37, 2661-2678. https://doi.org/10.1093/molbev/msaa120

81. Love RR, Steele AM, Coulibaly MB, Traore SF, Emrich SJ, Fontaine MC, Besansky NJ. Chromosomal inversions and ecotypic differentiation in *Anopheles gambiae*: the perspective from whole-genome sequencing. Mol Ecol. 2016 Dec;25(23):5889-906. https://doi.org/10.1111/mec.13888

82. Exposito-Alonso M, Vasseur F, Ding W, Wang G, Burbano HA, Weigel D. Genomic basis and evolutionary potential for extreme drought adaptation in *Arabidopsis thaliana*. Nat Ecol Evol. 2018 Feb;2(2):352-8., https://doi.org/10.1038/s41559-017-0423-0

83. Hancock AM, Brachi B, Faure N, Horton MW, Jarymowycz LB, Sperone FG, Toomajian C, Roux F, Bergelson J. Adaptation to climate across the *Arabidopsis thaliana* genome. Science. 2011 Oct 7;334(6052):83-6.https://doi.org/10.1126/science.1209244

84. Zhen Y, Harrigan RJ, Ruegg KC, Anderson EC, Ng TC, Lao S, Lohmueller KE, Smith TB. Genomic divergence across ecological gradients in the Central African rainforest songbird (*A ndropadus virens*). Mol. Ecol. 2017 Oct;26(19):4966-77.https://doi.org/10.1111/mec.14270

85. Ahrens CW, Byrne M, Rymer PD. Standing genomic variation within coding and regulatory regions contributes to the adaptive capacity to climate in a foundation tree species. Mol Ecol. 2019 May;28(10):2502-16. https://doi.org/10.1111/mec.15092

86. Miller AD, Hoffmann AA, Tan MH, Young M, Ahrens C, Cocomazzo M, Rattray A, Ierodiaconou DA, Treml E, Sherman CD. Local and regional scale habitat heterogeneity contribute to genetic adaptation in a commercially important marine mollusc (*Haliotis rubra*) from southeastern Australia. Mol. Ecol. 2019 Jun;28(12):3053-72. https://doi.org/10.1111/mec.15128

87. Crosbie PR, Boyce WM, Rodwell TC. DNA sequence variation in *Dermacentor hunteri* and estimated phylogenies of *Dermacentor* spp.(Acari: Ixodidae) in the New World. J. Med. Entomol. 1998 May 1;35(3):277-88. https://doi.org/10.1093/jmedent/35.3.277

88. Norris DE, Klompen JS, Keirans JE, Black IV WC. Population genetics of *Ixodes scapularis* (Acari: Ixodidae) based on mitochondrial 16S and 12S genes. J. Med. Entomol.1996 Jan 1;33(1):78-89. https://doi.org/10.1093/jmedent/33.1.78

89. Qiu WG, Dykhuizen DE, Acosta MS, Luft BJ. Geographic uniformity of the Lyme disease spirochete (*Borrelia burgdorferi*) and its shared history with tick vector (*Ixodes scapularis*) in the northeastern United States. Genetics. 2002 Mar 1;160(3):833-49. https://doi.org/10.1093/genetics/160.3.833

90. Rich SM, Caporale DA, Telford 3rd SR, Kocher TD, Hartl DL, Spielman A. Distribution of the *Ixodes ricinus*-like ticks of eastern North America. Proceedings of the National Academy of Sciences. 1995 Jul 3;92(14):6284-8. https://doi.org/10.1073/pnas.92.14.6284

91. de la Fuente J, Almazán C, Van Den Bussche RA, Bowman J, Yoshioka JH, Kocan KM. Characterization of genetic diversity in *Dermacentor andersoni* (Acari: Ixodidae) with body size and weight polymorphism. Exp. Parasitol. 2005 Jan 1;109(1):16-26. https://doi.org/10.1016/j.exppara.2004.10.004

92. Caporale DA, Rich SM, Spielman A, Telford SR, Kocher TD. Discriminating between *Ixodes* ticks by means of mitochondrial DNA sequences. Mol. Phylogenet. Evol. 1995 Dec 1;4(4):361-5. https://doi.org/10.1006/mpev.1995.1033

93. Mitani H, Takahashi M, Masuyama M, Fukunaga M. *Ixodes philipi* (Acari: Ixodidae): phylogenetic status inferred from mitochondrial cytochrome oxidase subunit I gene sequence comparison. J. Parasitol. Res. 2007 Jun;93(3):719-22. https://doi.org/10.1645/ge-953r.1

94. Anderson JF, Magnarelli LA. Biology of ticks. Infectious disease clinics of North America. 2008 Jun 1;22(2):195-215.

95. Hill CA, Wikel SK. The *Ixodes scapularis* Genome Project: an opportunity for advancing tick research. Trends Parasitol.. 2005 Apr 1;21(4):151-3.https://doi.org/10.1016/j.pt.2005.02.004

96. Van Zee JP, Geraci NS, Guerrero FD, Wikel SK, Stuart JJ, Nene VM, Hill CA. Tick genomics: the *Ixodes* genome project and beyond. Int. J. Parasitol. 2007 Oct 1;37(12):1297-305. https://doi.org/10.1016/j.ijpara.2007.05.011

97. Jongejan F, Nene V, de la Fuente J, Pain A, Willadsen P. Advances in the genomics of ticks and tick-borne pathogens. Trends Parasitol. 2007 Sep 1;23(9):391-6. https://doi.org/10.1016/j.pt.2007.07.004

98. Gray JS. Biology of Ixodes species ticks in relation to tick-borne zoonoses. Wiener Klinische Wochenschrift. 2002 Jul 1;114(13-14):473-8.

99. Parola P, Paddock CD. Travel and tick-borne diseases: Lyme disease and beyond. Travel Med Infect Dis. 2018;26:1-2. https://doi.org/10.1016/j.tmaid.2018.09.010

Supporting information

S1 Table. Summary of *Ixodes ovatus* and *Haemaphysalis flava* collected from the different locations of Niigata Prefecture and its corresponding sample number and number of *Rickettsia* infection per site

S2 Table. The difference in *Rickettsia* infection rates in *H. flava* haplotype groups and a table showing the distribution of *Rickettsia*-infected and uninfected *H. flava* in each genetic groups as shown in S2 Figure *(H. flava* median-joining network). The results of the z-score test for two populations proportions at $p < 0.05$ showed no significant difference between the *Rickettsia* detection rate in haplotype groups 1 and 2. The Welch t-test at $p < 0.05$ revealed no significant difference in the mean altitude of the two groups.

S1 Fig. Phylogenetic tree from the BEAST analysis of 59 haplotype *cox1* sequences of *I. ovatus.* The blue-labeled haplotypes indicate the presence of *Rickettsia* infection. The red parentheses provide the number of *Rickettsia*-positive individuals per haplotype. The black labeled haplotypes are negative for *Rickettsia* infection.

S2 Fig. Median-joining network of the 66 *cox1* haplotype sequences of *Rickettsia* positive and negative *H. flava.* Haplotype groups are indicated as 1 and 2.

S3 Fig. Phylogenetic tree from the BEAST analysis of 66 haplotype *cox1* sequences of *H. flava.* The blue-labeled haplotypes indicate *Rickettsia* infection in individual samples. The parentheses in red provide the number of *Rickettsia*-infected ticks. The black-labeled haplotypes are negative for *Rickettsia* infection.

Figure 1

Click here to access/download;Figure;Figure 1.TIF

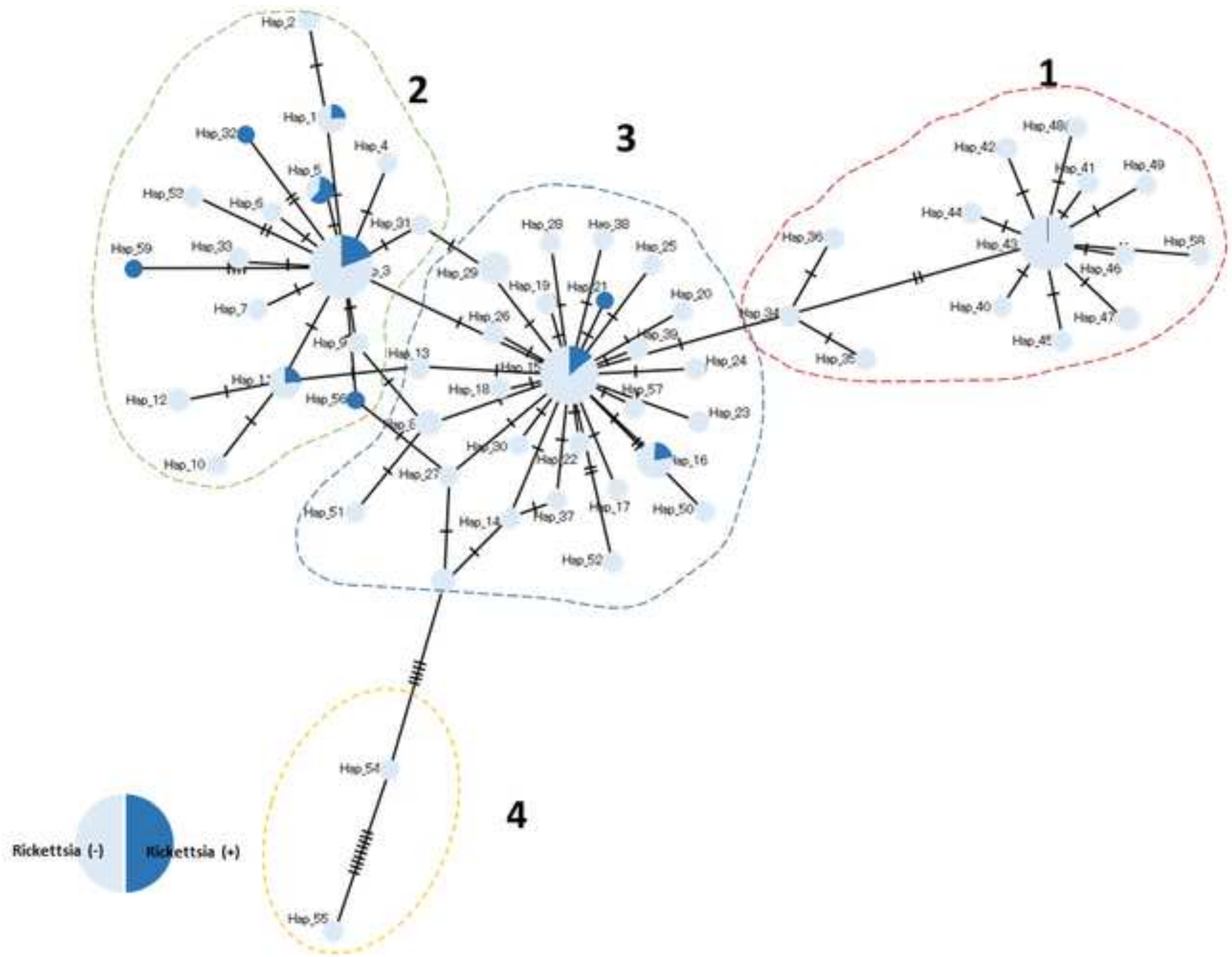

Figure 2

Click here to access/download;Figure;Figure 2.png

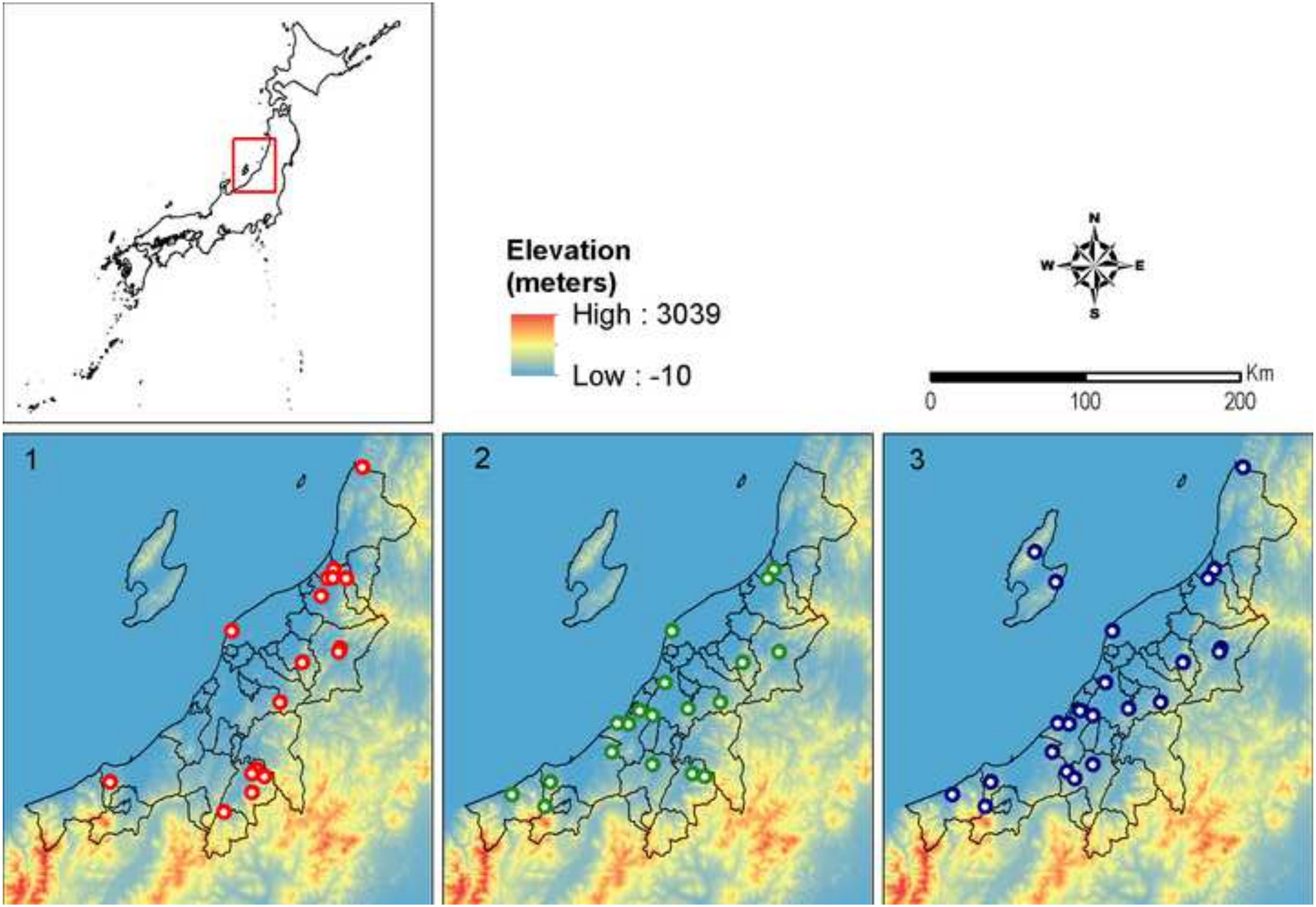

Figure 3

Click here to access/download;Figure;Figure 3.jpg

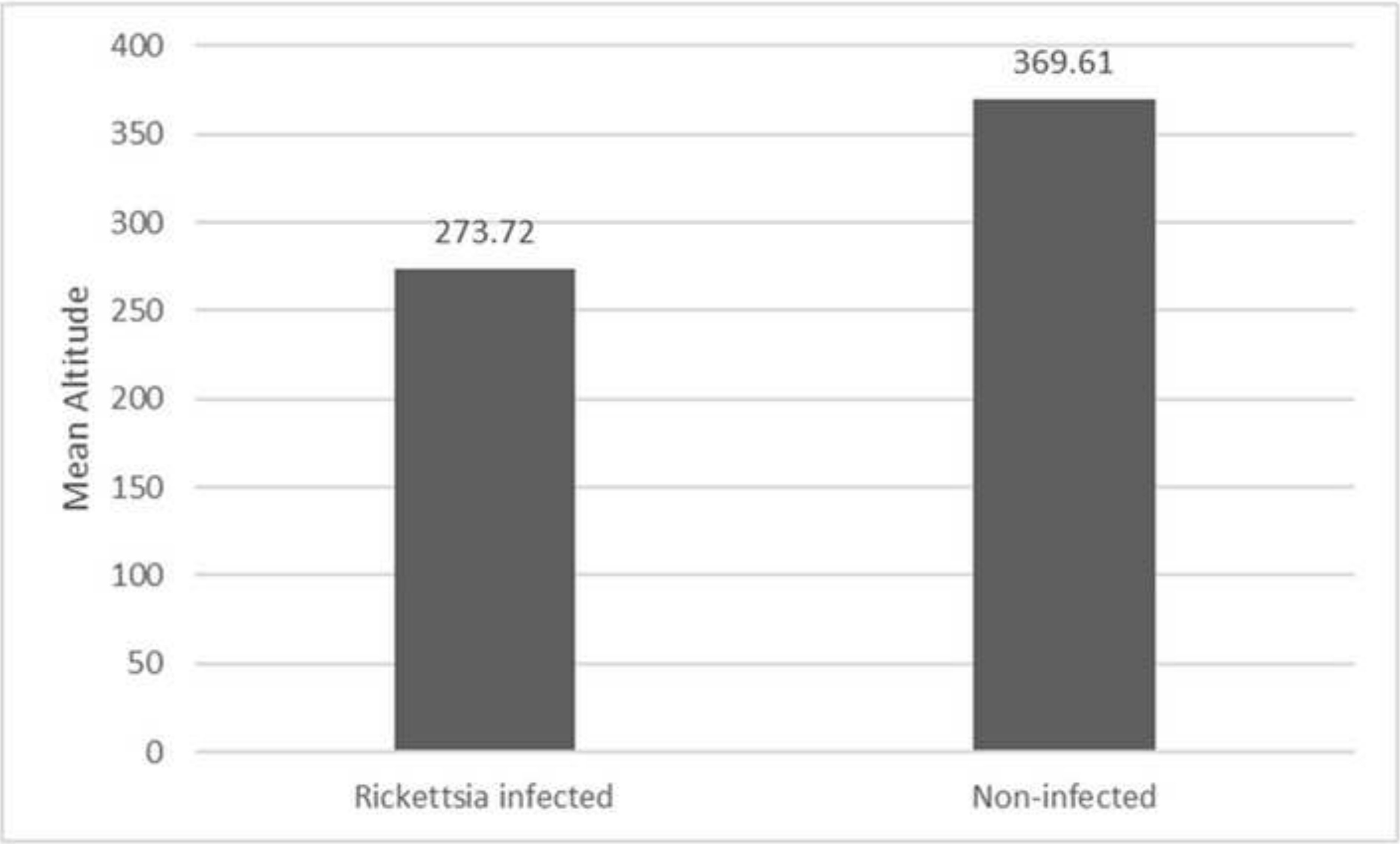

Figure 4

Click here to access/download;Figure;Figure 4.jpg

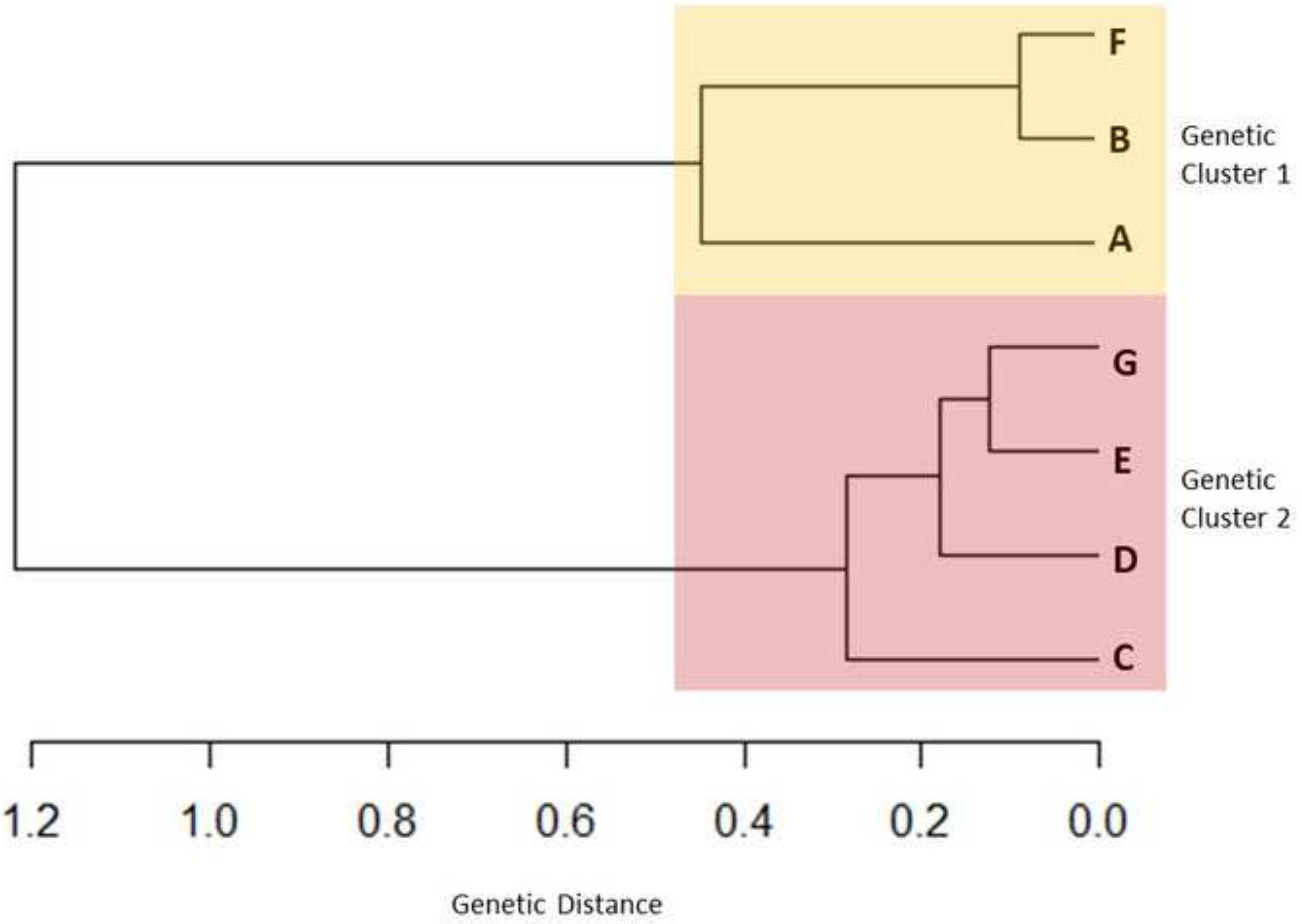

S1 Figure

Click here to access/download
Supporting Information
S1 Figure.docx

S2 Figure

Click here to access/download
Supporting Information
S2 Figure.docx

S2 Table

Click here to access/download
Supporting Information
S2 Table.docx

S3 Figure

Click here to access/download
Supporting Information
S3 Figure.docx

S1 Table

Click here to access/download
Supporting Information
S1 Table.xlsx
